# The role of 3D culture models and advanced chromatography in exosome research for triple-negative breast cancer

**DOI:** 10.1186/s43046-025-00322-x

**Published:** 2025-09-27

**Authors:** Mujibullah Sheikh, Harpritkaur Bagga, Yukta Bhojwani, Umesh Telrandhe

**Affiliations:** Department of Pharmaceutics, Datta Meghe College of Pharmacy, Datta Meghe Institute of Higher Education & Research (Deemed to be University), Wardha, Maharashtra 442001 India

**Keywords:** Triple-negative breast cancer (TNBC), Exosome small extracellular vesicles (sEVs), 3D culture models, Tumor microenvironment, Chromatographic techniques

## Abstract

Triple-negative breast cancer (TNBC) is among the most difficult subtypes of breast cancer to treat and is characterized by the absence of estrogen receptor, progesterone receptor, and HER2 expression. Owing to its Limited molecular targets and high degree of tumor heterogeneity, TNBC is associated with poor prognosis and restricted treatment options. Exosome small extracellular vesicles secreted by virtually all cell types have gained renewed attention for their role in tumor progression, metastasis, immune modulation, and drug resistance in TNBC. These vesicles carry biologically active cargo such as proteins, RNAs, Lipids, and metabolites that reflect the molecular state of their parent cells and facilitate intercellular communication. However, conventional 2D culture systems and classical exosome isolation methods fail to replicate the complexity of the tumor microenvironment and the diversity of exosomal populations. This review summarizes recent advances in the integration of three-dimensional (3D) culture systems and advanced chromatographic techniques to enhance the isolation, profiling, and functional analysis of TNBC-derived exosomes. We highlight the benefits of using 3D models, improvements in analytical workflows, and interdisciplinary approaches that are enabling progress in biomarker discovery, understanding therapy resistance, and developing exosome-based therapeutic strategies. By bridging technological innovation with biological insight, this review aims to support future advances in exosome research relevant to TNBC.

**Supplementary Information:**

The online version contains supplementary material available at 10.1186/s43046-025-00322-x.

## Introduction

Triple-negative breast cancer (TNBC) presents a formidable challenge in oncology because of its lack of hormone receptor and HER2 expression, which precludes targeted therapies and often leads to progression and poor prognosis. In exosome research, three-dimensional (3D) culture models have been heralded for their ability to recapitulate the in vivo tumor microenvironment, offering physiologically relevant cell–cell and cell–matrix interactions that profoundly influence exosome yield, cargo composition, and functional phenotypes compared with those of conventional two-dimensional systems [1]. However, these models introduce considerable variability in exosome isolation workflows, as matrix-derived contaminants and variable growth-nutrient gradients can complicate reproducibility and downstream analyses. Complementing biological models, advanced chromatography techniques—most notably size-exclusion chromatography (SEC), affinity chromatography, and ion-exchange methods—have substantially improved the purity and integrity of isolated exosomes, minimizing shear-induced vesicle damage inherent to ultracentrifugation and reducing protein contamination from complex biological fluids [2]. However, chromatographic approaches face their own trade-offs: SEC often sacrifices total yield for enhanced purity and requires large input volumes, whereas affinity- and ion-exchange methods can be cost-prohibitive at scale and may bias vesicle subpopulations through selective ligand interactions [3]. Critically, the integration of 3D culture systems with state-of-the-art chromatographic protocols demands rigorous standardization to balance the fidelity of tumor-derived exosome profiles against the practical constraints of isolation efficiency, purity, and scalability, underscoring the need for harmonized methodological frameworks in TNBC exosome research [4].

**Fig. 1:**
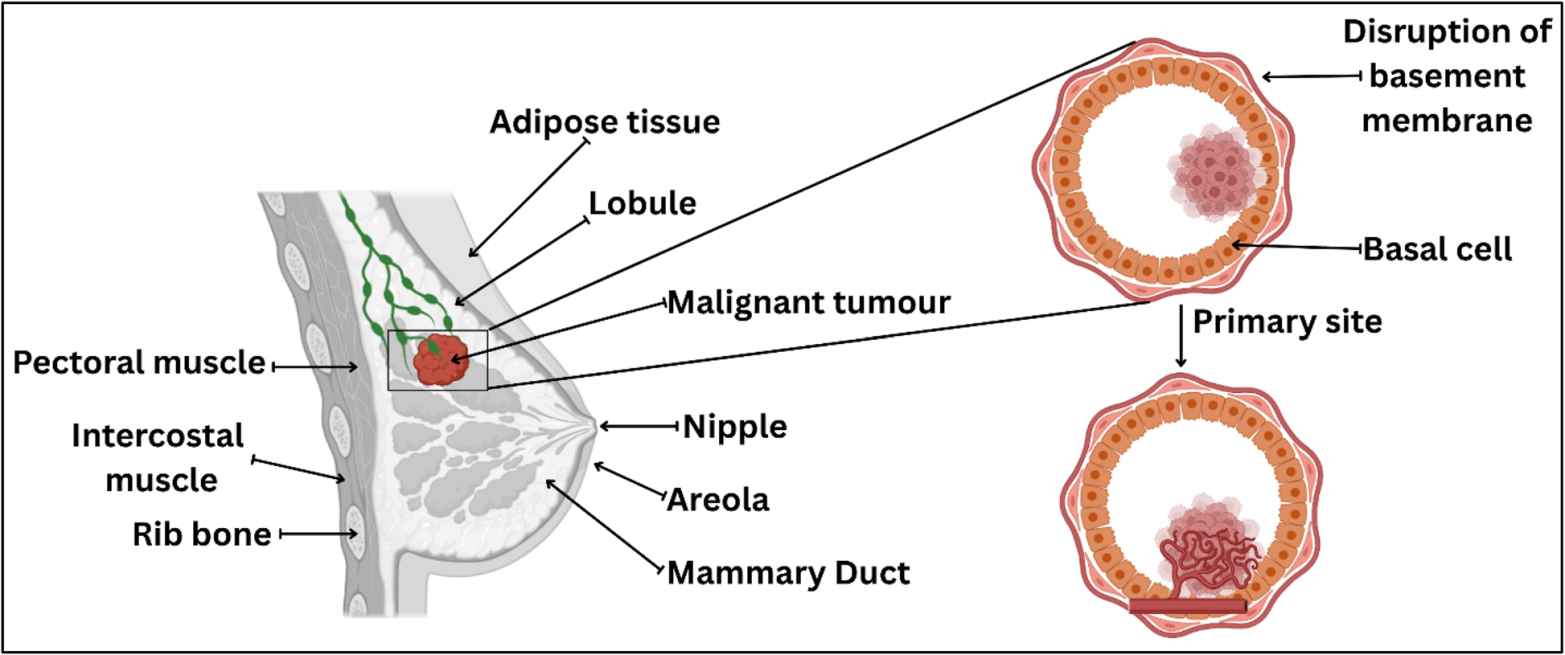
Breast cancer pathogenesis and metastasis: overview breast cancer typically develops in the cells of the mammary ducts (ductal carcinoma) or the lobules (lobular carcinoma), which represent the primary categories of this disease. As cancer progresses, malignant cells can infiltrate surrounding healthy breast tissue and may metastasize, most often spreading to lymph nodes in the underarm (axillary) region. In more advanced cases, cancerous cells can reach additional lymph nodes or distant organs. However, some breast cancers remain confined to the breast tissue and do not spread beyond their original site. Created in BioRender

Breast cancer has the highest incidence rate among all cancers affecting women worldwide and remains a leading cause of cancer-related deaths in this population. Figure 1 provides a visual overview of breast anatomy and highlights key aspects of malignant breast cancer pathophysiology, offering an essential context for understanding disease progression. Over one-fourth of cancers in women in 2022 were breast cancers [5]. Triple-negative breast cancer (TNBC), which accounts for approximately 15–20% of all breast cancer cases, is a highly aggressive subtype characterized by rapid progression, early recurrence, and poor prognosis due to the absence of estrogen, progesterone, and HER2 receptors, making it difficult to treat with conventional targeted therapies [6]. This molecular profile contributes to the high heterogeneity, rapid progression, early recurrence, and overall poor prognosis of TNBC [7].

Since actionable molecular targets are lacking, the cornerstone remains chemotherapy, with a prominent use being platinum agents or combination therapy [8]. However, the efficacy of these conventional methods is invariably plagued by excessive systemic toxicity, drug resistance, and a lack of selectivity for cancerous cells. Thus, novel therapeutic methods and drug delivery systems tailored to improve treatment efficacy without exacerbating side effects in TNBC patients are urgently needed.

In this context, small extracellular vesicles (sEVs), especially exosomes, have attracted increasing attention because of their potential utility in cancer diagnosis and treatment. sEVs are nanoscale (< 200 nm) membrane-shrouded vesicles released from virtually any type of cell [9] and are found in numerous body fluids, such as blood, urine, breast milk, and saliva. These proteins are produced from the endosomal pathway and are secreted outside in response to the fusion of multivesicular bodies with the plasma membrane [10]. They are packed with a myriad of cargo, ranging from mRNAs, microRNAs, noncoding RNAs, proteins, DNA fragments, and lipids to metabolites, enabling them to facilitate local and systemic intercellular communication [11].

sEVs are implicated in a variety of tumor development processes, including the proliferation of cancer cells, angiogenesis, evasion from the immune system, and metastasis, most importantly by interacting with the tumor microenvironment in a dynamic fashion. In TNBC, sEVs are likely to be critical in delineating the immunological environment, modifying the extracellular environment, and enhancing the metastatic capabilities of tumor cells [12]. Moreover, sEVs mirror the physiological and pathophysiological state of the cells from which they originate, rendering them very useful noninvasive markers for cancer detection, prognosis, and monitoring of therapeutic efficacy.

Small extracellular vesicles (sEVs), particularly exosomes, are nanoscale, membrane-bound particles secreted by virtually all cell types into body fluids such as blood, urine, and breast milk. These vesicles carry diverse molecular cargo—including mRNAs, microRNAs, noncoding RNAs, proteins, lipids, and metabolites—and play crucial roles in local and systemic intercellular communication [13]. In triple-negative breast cancer (TNBC), exosomes contribute to tumor progression by promoting proliferation, immune evasion, angiogenesis, and metastasis, largely through dynamic interactions with the tumor microenvironment. Their content reflects the physiological state of the parent cells, making them valuable noninvasive biomarkers for diagnosis, prognosis, and therapy monitoring. Moreover, exosomes possess natural advantages such as low immunogenicity, high biocompatibility, and the ability to traverse biological barriers, which position them as promising candidates for targeted drug delivery systems in TNBC therapy.

Despite promising data from preclinical tests, numerous challenges remain, including establishing methodologies to standardize exosome isolation, discovering absolute biomarkers for TNBC-derived sEVs, and ensuring scalability and safety for use in clinical settings. Nevertheless, development in this area is advancing at a rapid pace, with numerous studies aimed at determining how sEVs function in cancer immunotherapies, modulation of drug resistance, and carriers for drug combinations.

### Integrating 3D cell culture and chromatography in experimental design

Three-dimensional cell culture, which involves spheroids, organoids, and bioprinted tissues, betterates native tissue architecture, gradients, and function than flat monolayers do, but its analytical demands exceed those of classical 2D assays. Chromatography fills this gap by selectively separating, enriching, and quantifying low-abundance metabolites, proteins, and xenobiotics released by or retained within 3D systems. Recent breakthroughs knit these two technologies into seamless workflows: silk fibroin “stationary-matrix” columns that double as extracellular scaffolds for live cells [14]; “organ-in-a-column” liver organoids packed inside PTFE LC housings that feed drug-laden media directly to LC‒MS detectors without manual sampling [15]; FDA-validated self-cleaning LC‒MS plumbing that involves > 1000 injections of protein-rich organoid medium through on-line solid‒phase extraction and automated filtration‒back‒flush cycles; and 3D-printed microfluidic batch‒chromatography chips with microliter dead‒volumes that purify monoclonal antibodies produced in spheroids. These designs solve perennial challenges in maintaining perfusion and viability under low-shear flow, preventing column clogging with glass-bead spacers, taming matrix effects via short-chain C4 phases or acetonitrile-rich gradients, and miniaturizing hardware to match scarce sample volumes (≤ 2.5 µL) from single spheroids monitored by NMR or LC-HRMS. In metabolomics, ultrahigh-performance LC-HRMS of colorectal cancer-adipocyte coculture secretomes revealed 105 discriminatory molecules, including lipid mediators of tumor-adipose cross-talk [14]; targeted ^13C-tracer LC‒MS in 3D cancer spheroids resolved pathway fluxes unavailable in 2D [14]; and radial slice-selective NMR coupled with Abel-inverted diffusion profiles mapped lactate accumulation across spheroid cores, validating chromatographic readouts [16]. Proteomic pipelines now routinely harvest Hundreds of replicate spheroids from rotating-wall bioreactors, solubilize them in surfactant cocktails, and inject tryptic digests onto nano-LC columns, yielding thousands of protein identifications per run. Downstream bioprocess engineers exploit affinity, ion exchange, and hydrophobic interaction modes in tandem to purify secreted therapeutic proteins from 3D culture supernatants at the bench scale, guided by tag‒ligand pairs cataloged for rapid capture and elution [17]. Together, these converging advances transform experimental design: chromatography becomes not a post hoc assay but an integrated, programmable component of dynamic 3D culture platforms, enabling high-throughput drug metabolism screens, biomarker discovery, and continuous product capture while preserving the complex biology that makes 3D models indispensable.

### Molecular heterogeneity and genetic drivers

Triple-negative breast cancer (TNBC) has deep Genomic heterogeneity that extends far beyond its clinical diagnosis, creating major obstacles for successful therapy. Genomic and transcriptomic analyses have stratified TNBC into distinct molecular subtypes, most commonly including the basal-like 1 (BL1), basal-like 2 (BL2), mesenchymal (M), immunomodulatory (IM), and luminal androgen receptor (LAR) subtypes, each characterized by unique gene expression patterns and biological behaviors [18]. Tumors with BL1 features have fast growth rates and use DNA damage repair paths, which makes them react better to platinum-based chemotherapy. In contrast, the growth factor signaling and myoepithelial pathways are more active in BL2 tumors, whereas the M and MSL subtypes have greater expression of epithelial‒mesenchymal transition and growth factor pathways, although MSL cells are less likely to divide [19]. The IM subtype is defined by heightened immune signaling and expression of immune-related genes, whereas the LAR subtype is driven by androgen receptor signaling and luminal gene expression, setting it apart from the basal-like subtypes [20].

Genetic changes in TNBC include frequent mutations in TP53 (up to 80%) and PIK3CA (approximately 10%) and the removal of BRCA1/2 function (approximately 20%), the latter connecting cancer to PARP inhibitor sensitivity [21–23]. More genomic changes, such as extra copies (e.g., 1q and 8q) and rearrangements (including NOTCH and MAST kinase fusions), further contribute to the complexity of the disease and impact the therapeutic response [24]. Since different groups of molecules are involved, treatment success can vary; basal-like subtypes, especially BL1, generally respond better to first-line therapy, and this type of subtype has a more favorable prognosis; however, the mesenchymal and LAR subgroups are often less responsive to treatment and have poorer outcomes. Therefore, the detailed molecular and genetic features of TNBC reveal why we need to classify patients better and offer tailored treatments to achieve better results [25].

### Hydrogel scaffolds in TNBC

Hydrogel scaffolds have emerged as a vital component of 3D culture systems for triple-negative breast cancer (TNBC), providing a physiologically relevant microenvironment that closely mimics the tumor extracellular matrix (ECM) [26, 27]. Scaffolds such as Matrigel and Cultrex provide TNBC cells with ECM proteins such as laminin and collagen IV, as well as entactin and heparan sulfate proteoglycans, which help the cells grow, invade, and respond to drugs [28].

Compared with 2D model cells, TNBC cells cultured in hydrogels exhibit greater spheroid integrity, enhanced proliferation, and altered drug sensitivity [29]. For example, MDA-MB-231 cells formed larger, more compact spheroids with increased circularity and Ki-67 expression when embedded in 2.5% Matrigel, as opposed to those cultured without hydrogels, which produced only loose aggregates and smaller structures. Despite these advantages, standard hydrogels such as Matrigel face challenges such as batch-to-batch variability and limited tunability in mechanical stiffness, which are critical for studying mechanotransduction in TNBC progression [30].

To overcome these limitations, novel biomimetic hydrogels such as EKGel have been developed. EKGels feature enhanced covalent crosslinking and nanofibrillar structures, allowing precise control of stiffness and better replication of the TNBC ECM, supporting long-term culture and maintaining tumor heterogeneity [31]. As such, hydrogel-based platforms are increasingly instrumental in drug screening, mechanobiology studies, and the development of precision therapies for aggressive breast cancer subtypes such as TNBC [32].

### Batch-to-batch variability of 3D culture matrices

Batch-to-batch variability is a significant Limitation in the use of biologically derived 3D culture matrices such as Matrigel and natural hydrogels. These matrices, sourced from animal tissues, inherently possess ill-defined and variable compositions between production lots, leading to inconsistencies in experimental conditions and substantial issues for reproducibility. The protein content, growth factor concentration, and mechanical properties can vary across batches, directly affecting cell growth, differentiation, morphology, and experimental outcomes [33].

Such variability impacts the reliability and robustness of 3D in vitro models, particularly for drug screening, high-throughput assays, or mechanistic studies where subtle environmental cues can alter cell behavior. Variation in matrix composition may introduce assay noise, complicate interpretation of data, and mask true biological effects. This is especially problematic when comparing results across laboratories or over time, as batch-specific differences can confound biological conclusions and undermine confidence in translational relevance [34].

The consequences are not Limited to assay variability. Automation and scalability are hampered by the need for strict quality control and batch testing. Synthetic hydrogels offer a partial solution, allowing for greater standardization and reproducibility, but they may lack the full biological complexity of natural matrices. Regardless, ensuring consistency in extracellular matrix composition remains critical for advancing the reliability of 3D culture platforms. Until more standardized or customizable matrices are widely available, careful batch tracking, validation, and transparency in reporting matrix characteristics are essential for ensuring scientific rigor [35].

### Rationale for review

Triple-negative breast cancer (TNBC) remains one of the most challenging forms of breast cancer due to its aggressive nature, high heterogeneity, and lack of effective targeted therapies. Conventional treatment strategies, which are largely reliant on chemotherapy, are hindered by issues such as systemic toxicity, drug resistance, and poor selectivity for cancer cells, resulting in suboptimal patient outcomes. The urgent need for novel diagnostic and therapeutic approaches is underscored by the increasing incidence and mortality rates associated with TNBC, especially in regions such as India, where breast cancer accounts for a significant proportion of cancer cases and deaths among women.

Recent advances in cancer biology have highlighted the pivotal role of small extracellular vesicles (sEVs), particularly exosomes, in the progression, metastasis, and drug resistance of TNBC. Exosomes, owing to their unique molecular cargo and ability to mediate intercellular communication, have emerged as promising noninvasive biomarkers for cancer diagnosis, prognosis, and therapeutic monitoring. Their stability in biological fluids and specificity to their cell of origin make them attractive candidates for liquid biopsy-based diagnostics and targeted drug delivery systems.

However, traditional 2D cell culture models and classical exosome isolation techniques are Limited in replicating the complexity of the tumor microenvironment and the heterogeneity of exosome populations. The advent of advanced 3D culture models and state-of-the-art chromatographic methods has opened new avenues for more accurately studying exosome biology, enabling the discovery of novel biomarkers and the development of more effective therapeutic strategies. These interdisciplinary approaches are crucial for unraveling the molecular underpinnings of TNBC, understanding the mechanisms of therapy resistance, and advancing exosome-based medicine.

Given the rapid evolution of this field, a comprehensive review is warranted:

- Summarize the current understanding of exosome biology in TNBC.
- The Limitations of conventional methodologies and the advantages offered by 3D culture models and advanced chromatographic techniques are highlighted.
- The translational potential of exosome research for improving TNBC diagnosis, prognosis, and therapy is discussed.
- Identify ongoing challenges and future directions for integrating technological innovations with biological insights in exosome research.

By synthesizing recent findings and methodological advances, this review aims to provide a critical resource for researchers and clinicians seeking to leverage exosome science for better management of TNBC1.

## Exosomes in TNBC

Triple-negative breast cancer (TNBC) is a subtype characterized by the absence of estrogen receptor, progesterone receptor, and HER2 expression, leading to an aggressive tumor phenotype and limited treatment options [36]. Exosomes—a subset of small extracellular vesicles (sEVs) that are typically 30–150 nm in size—have emerged as critical mediators of cancer biology. They originate from the endosomal pathway and carry molecular cargo such as proteins, RNAs, lipids, and DNA fragments, which reflect the physiological or pathological state of their parent cells [37, 38]. In TNBC, exosomes actively participate in intercellular communication, promoting invasion, metastasis, chemoresistance, and immune modulation [39]. The following sections explore the role of exosomes in TNBC by focusing on their diagnostic and prognostic potential, their role in epithelial–mesenchymal transition (EMT), and their utility as molecular fingerprints of originating cells [21].

### Exosomes carry signature markers from their cells of origin

Exosomes encapsulate a specific and regulated set of biomolecules that convey the identity and status of their parent cells. Rather than being random waste products, their cargo includes signature proteins (e.g., tetraspanins CD9, CD63, and CD81), lipids, RNAs, and sometimes DNA, which are tightly regulated by the donor cell’s biogenesis machinery [38, 40]. This selective enrichment gives exosomes the capacity to serve as molecular fingerprints, indicating both their cellular identity and health status, making them highly suitable for liquid biopsy-based diagnostics [41]. Moreover, exosomes derived from TNBC cells are molecularly distinct from those derived from normal or other tumor types because of the presence of specific microRNAs and signaling molecules involved in oncogenesis and metastasis [38, 42]. Proteomic analyses have validated this unique feature, revealing both overlaps with established exosome databases and exclusive vesicle-specific proteins [43]. These molecular signatures support early disease detection and therapeutic monitoring, and advances in tools such as cryo-electron microscopy and single-vesicle profiling have improved exosome characterization and engineering for therapeutic use [44]. Furthermore, breakthroughs in high-resolution techniques such as cryoelectron microscopy and single-exosome analysis have enhanced the ability to isolate, characterize, and even engineer exosomes, thereby paving the way for potential applications of exosomes as targeted delivery vehicles and therapeutic agents [45]. Exosome stability within biological fluids enhances their detectability and biomarker potential. Furthermore, the mechanism of selective internalization by recipient cells, predicated upon receptor‒ligand interactions, unequivocally substantiates their functional importance in both physiological and pathological contexts [41]. In summary, the empirical data presented in the scientific literature provide substantial corroboration for the hypothesis that exosomes function as carriers of specific biomolecular markers that accurately denote their originating cells. This characteristic is exploited for the advancement of novel diagnostic and therapeutic strategies within the realm of precision medicine, concurrently constituting a significant focal point for ongoing research endeavors [38].

### Exosomes for the diagnosis of TNBC

**Fig. 2:**
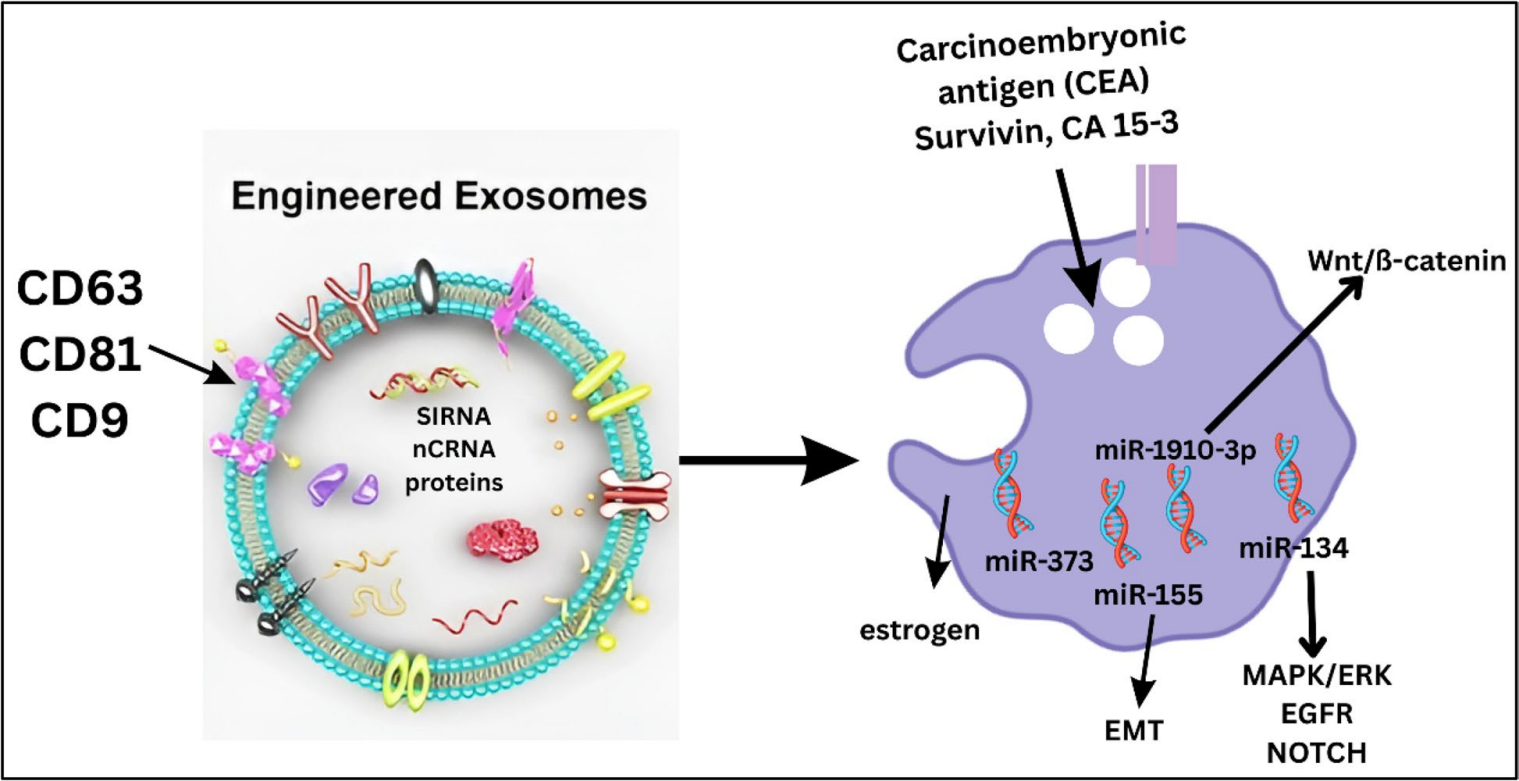
Exosomes as biomarkers for triple-negative breast cancer (TNBC): The figure illustrates the diverse protein and microRNA (miRNA) contents within exosomes, highlighting their role in the identification and diagnosis of TNBC through liquid biopsy. Key exosomal biomarkers include carcinoembryonic antigen (CEA), survivin, cancer antigen 15–3 (CA 15–3), miR-373, miR-134, miR-1910-3p, and miR-155, indicating potential for noninvasive cancer detection and monitoring. Created in BioRender

In TNBC diagnostics, exosomes enable noninvasive molecular profiling through liquid biopsy techniques. Their content mirrors tumor biology more accurately than traditional biopsies do by capturing tumor heterogeneity and dynamic microenvironmental changes [41, 46]. In triple-negative breast cancer (TNBC), the exosomal content within patient biofluids significantly differs from that of their nonmalignant counterparts, thereby presenting a noninvasive modality for early diagnostic evaluation, disease surveillance, and prognostic assessment [42]. Exosomes found in TNBC patient fluids carry protein biomarkers such as carcinoembryonic antigen (CEA), survivin, and CA 15–3, all of which are correlated with disease presence and severity [47]. The detection of these proteins not only facilitates diagnostic confirmation but also yields critical insights into disease staging and prospective tumor advancement, considering that such biomarkers are frequently associated with a more aggressive tumor phenotype [42]. As illustrated in Fig. 2, engineered exosomes carrying proteins and microRNAs, such as miR-373, miR-134, miR-155, and miR-1910-3p, can modulate key signaling pathways such as the Wnt/β-catenin, MAPK/ERK, EGFR, and NOTCH pathways, enhancing the specificity of TNBC diagnosis [48].

For example, miR-1910-3p targets MTMR3 and activates prometastatic pathways, making it a promising early detection biomarker. Exosomes thus provide clinicians with a dynamic, multidimensional view of disease status, treatment response, and potential resistance development [42, 46]. This method supports the development of a diagnostic tool that integrates proteomic and transcriptomic data to guide prognosis and therapy, extending beyond the mere identification of genetic anomalies to encompass proteomic and transcriptomic variations, which collectively elucidate disease prognosis. In summary, exosomal profiling via liquid biopsy for triple-negative breast cancer (TNBC) has emerged as a pioneering strategy, transforming the specificity provided by cellular protein markers, including carcinoembryonic antigen (CEA), survivin, and cancer antigen 15–3 (CA 15–3), into regulatory insights afforded by miRNAs such as miR-373 and miR-1910-3p. Consequently, this approach yields a more sensitive and comprehensive methodology for early detection, strategic therapeutic guidance, and monitoring of neoplastic progression in TNBC [42, 47].

Despite burgeoning interest in exosome-based biomarkers for triple-negative breast cancer (TNBC), substantial inconsistencies hinder their clinical translation. Exosomal cargo, including miRNAs, proteins, and lipids, shows marked heterogeneity both between patients and within individual tumors [49]. This variability is driven by the genetic and phenotypic diversity of TNBC subclones, each releasing vesicles with distinct molecular profiles that may not be reproducible across different patient samples or studies. Despite these problems, methodological differences in exosome isolation, purification, and characterization can yield widely divergent results even from the same biological source, as contaminants such as nonexosomal particles are often coisolated [50]. These factors complicate the establishment of a reliable, universal set of TNBC-derived exosomal biomarkers with strong diagnostic or prognostic value. Therefore, the current landscape is characterized by frequent discordance between discovery studies, limited cross-cohort validation, and variable assay performance. This underscores the critical need for harmonized protocols, rigorous validation in diverse patient populations, and comprehensive reporting standards to ensure that exosomal biomarker research can move toward meaningful clinical application [51].

### Exosomes initiate epithelial mesenchymal transition in breast cancer and TNBC, influencing metastasis to other organs

Exosomes have been shown to be the key driving agents that trigger epithelial‒mesenchymal transition (EMT) in breast cancer and, as a result, play vital roles in metastatic dissemination to distant organs in both breast cancer and triple-negative breast cancer (TNBC) [52]. These nanosized extracellular vesicles contain a diverse repertoire of bioactive cargo, including microRNAs (miRNAs), long noncoding RNAs (lncRNAs), circular RNAs (circRNAs), proteins, and lipids. These components facilitate the modulation of gene expression and signal transduction, promoting the epithelial‒mesenchymal transition (EMT) associated with enhanced aggressiveness, primarily through the action of regulatory or suppressor cells, while also possessing intrinsic capabilities for executing cellular reprogramming processes [52]. EMT is a process in which epithelial cells acquire mesenchymal traits, enabling increased motility and invasiveness, which is possible only by reprogramming the genome. This reprogramming is the result of the concerted action of some key proteins for cell nuclei, such as β‐catenin, TWIST, SNAIL2, SLUG, ZEB1, and ZEB2, and it can eliminate the characteristics of epithelial cells, such as E-cadherin, while it exchanges them with mesenchymal ones, the most typical of which are vimentin and N-cadherin; finally, the cells become capable of penetrating into the tissue and enabling the dissemination of diverse oncogenic signals. The upregulation of mesenchymal-like genes in tumors is indicative of the evolutionary presence of cells with invasive and migratory capacities [53]. These regulatory mechanisms and their impacts on TNBC are summarized in Table 1. While altering these transcription factors alone is not sufficient to completely abrogate EMT, exosomes facilitate intercellular communication that additionally reinforces the EMT process by regulating the transcriptional machinery at multiple levels [54].

**Table 1:** Key factors that influence epithelial‒mesenchymal transition (EMT) and their impact on TNBC

Key factor	Category	Mechanism of action	Impact on TNBC	Ref
Snail, slug, twist, ZEB1/2	EMT transcription factors	Repress epithelial markers (e.g., E-cadherin) and induce mesenchymal gene expression; interact with miRNAs in feedback loops	Enhance cellular plasticity, invasiveness, metastasis, and chemoresistance	[55]
FOXC1 and MSX2	EMT-associated transcription factors	Transcriptionally activate EMT programs; MSX2 mediates TNF-induced EMT	Associated with mesenchymal phenotype, tumor grade, metastasis, and poor prognosis	[56]
TGF-β signaling	Signaling pathway	Activates Smad-dependent and noncanonical (MAPK/ERK, PI3K/AKT) pathways	Induces EMT-TFs, stemness, chemoresistance, and metastasis	[57]
Wnt/β-catenin pathway	Signaling pathway	Ligand‒receptor activation stabilizes nuclear β-catenin, promoting mesenchymal gene expression	Promotes EMT, invasiveness, and chemoresistance	[56]
Notch signaling	Signaling pathway	Activated via ligand (Jagged/Delta) interaction; NICD translocation activates genes (Hes1, Hey1)	Drives EMT, proliferation, migration, and invasion in TNBC	[57]
NF-κB signaling	Signaling pathway	Cytokines (e.g., TNF-α) trigger NF-κB nuclear translocation and EMT gene activation	Supports EMT, inflammation-driven invasion, and tumor aggressiveness	[55]
miRNAs (e.g., miR-200 family, miR-203, miR-205)	Non-coding RNAs	Downregulate EMT-TFs (e.g., ZEB1/2) and maintain epithelial traits	Loss promotes EMT, stemness, and resistance; overexpression reverses EMT	[58]
Long noncoding RNAs (e.g., HOTAIR, linc-ZNF469-3)	Non-coding RNAs	Act as ceRNAs, modify chromatin, and interact with EMT-TFs	Promote EMT, invasion, and metastasis	[56, 59]
Epigenetic regulators (DNA methylation, histone modifications)	Epigenetic modifiers	Modify chromatin to regulate EMT genes and miRNAs	Enhance EMT, metastasis, and therapy resistance	[54, 56]
Tumor microenvironment (cytokines, chemokines, stromal cells)	Microenvironment components	Cytokines and ECM remodeling factors influence tumor cells via ligand‒receptor signaling	Stimulates EMT, stemness, and metastatic spread	[55]
Oncogenic kinases (e.g., PKMYT1)	Kinase-driven mechanisms	PKMYT1 activates Notch and dysregulates the cell cycle	Enhances proliferation, invasion, and poor prognosis	[60]
Extracellular matrix proteins and integrins	Structural/adhesion molecules	Alter ECM and integrin expression (e.g., αvβ3, claudins); promote cadherin switch	Increases motility, invasion, and chemoresistance	[55]

**Fig. 3:**
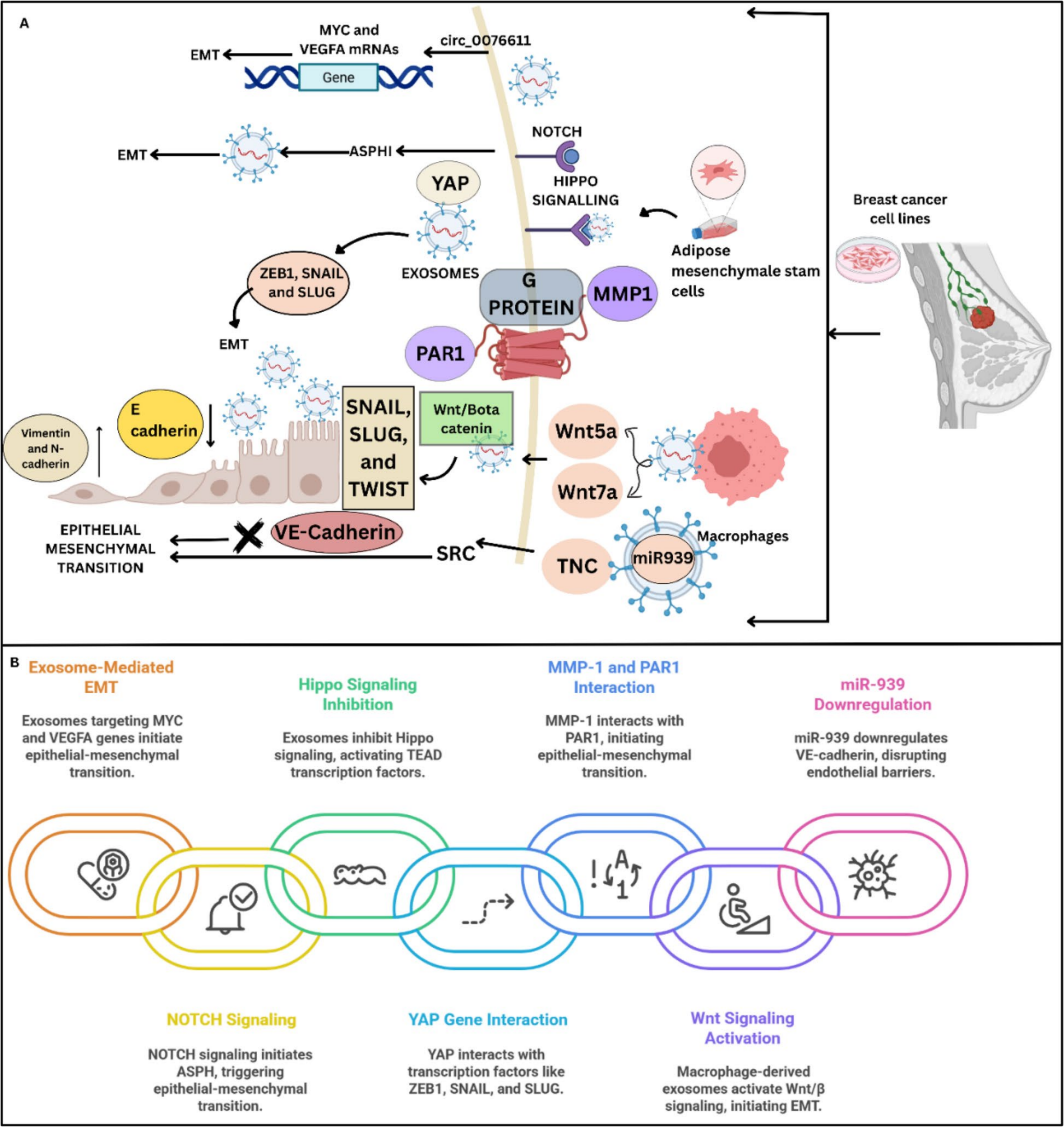
Exosome-mediated pathways driving epithelial–mesenchymal transition (EMT) and metastasis in breast cancer. **A** Schematic illustration of the complex signaling networks through which exosomes mediate epithelial–mesenchymal transition (EMT) in triple-negative breast cancer (TNBC) and other breast cancer subtypes. Exosomal circ_0076611 targets MYC and VEGFA mRNAs, triggering EMT. NOTCH signaling, via ASPH activation in breast cancer cells, also promotes EMT. Exosomes derived from adipose mesenchymal stem cells inhibit the Hippo pathway, thereby activating TEAD transcription factors through YAP/TAZ nuclear translocation, leading to the upregulation of mesenchymal markers (Vimentin and N-cadherin) and the downregulation of epithelial markers (E-cadherin). YAP activation cross-communicates with EMT-associated transcription factors such as ZEB1, SNAIL, and SLUG. MMP-1 binds to the G protein-coupled receptor PAR1, initiating further EMT signaling. Macrophage-derived exosomes containing Wnt5a and Wnt7a stimulate Wnt/β-catenin signaling, activating EMT-inducing transcription factors (SNAIL, SLUG, and TWIST). Additionally, exosomal miR-939 downregulates VE-cadherin, compromising endothelial integrity and facilitating tumor dissemination. **B** Summary of signaling pathways involved in breast cancer metastasis: exosome-mediated EMT, Hippo signaling inhibition, MMP-1/PAR1 interaction, miR-939 downregulation, NOTCH signaling, YAP gene interaction, and Wnt signaling activation. These interconnected pathways collectively contribute to breast cancer cell plasticity, migration, and systemic metastasis. Created with BioRender.com

The biosynthesis of exosomes within neoplastic cells is intricately associated with the activation of pivotal signaling pathways, including the Notch, Hippo, and Wnt/β-catenin pathways, which play crucial roles in the regulation of epithelial‒mesenchymal transition (EMT). For example, the interplay between Notch receptors and their ligands, such as jagged proteins, alongside regulatory enzymes such as ADAM10/17, in conjunction with aspartate beta-hydroxylase (ASPH)-mediated activation, culminates in the generation of prometastatic exosomes, thereby facilitating aggressive tumor progression [61]. Conversely, upon inactivation of the Hippo signaling cascade, YAP/TAZ undergoes dephosphorylation, facilitating its translocation to the nucleus via interaction with TEAD transcription factors. Consequently, YAP/TAZ transactivate the expression of numerous mesenchymal genes while concurrently repressing epithelial gene expression, an effect that is potentiated by exosomal release from mesenchymal stem cell-derived adipocytes [61]. Moreover, Wnt-bound exosomes with Wnt ligands, e.g., Wnt5a derived from macrophages and Wnt7a, encode the canonical β-catenin signaling pathway, which is vital for the stabilization of β-catenin and the transcriptional activation of EMT-related genes, thus enhancing cancer cell motility and guiding organ-specific colonization, such as that in the lung [62]. These interconnected signaling cascades and exosome-mediated EMT mechanisms are illustrated in Fig. 3.

In addition to protein- and ligand-based signals, exosomes facilitate the transfer of noncoding RNAs that significantly influence the modulation of epithelial‒mesenchymal transition (EMT) transcription factors. Exosomal microRNAs (miRNAs), including miR-9, miR-10b, and miR-155, specifically target messenger RNAs (mRNAs) encoding proteins essential for sustaining the epithelial phenotype. The aberrant expression of these genes is correlated with a transition toward a mesenchymal phenotype [62]. Furthermore, exosomes facilitate the transport of long noncoding RNAs (lncRNAs), such as MALAT1 and ID4, which are involved in the upregulation of oncogenic circular RNAs (circRNAs), such as circ 0076611, in triple-negative breast cancer (TNBC). These circRNAs interact with messenger RNAs (mRNAs) encoding MYC and VEGFA, thereby increasing cellular proliferation and migration and further promoting epithelial‒mesenchymal transition (EMT) [53, 63]. The interplay among these noncoding RNAs and associated signaling pathways, including the Notch and Hippo pathways, not only reinforces the EMT process but also endows breast cancer cells with increased stemness and chemoresistance [64].

The clinical implications of these biological processes have been elucidated by empirical studies indicating that exosomes derived from triple-negative breast cancer (TNBC) can modulate the tumor microenvironment, thereby promoting the establishment of metastatic niches within vital organs such as the brain and liver. This phenomenon is mediated via the transfer of integrins and other cell surface molecules that enhance organ-specific colonization [65]. Furthermore, nascent evidence indicates that engineered exosomes aimed at specific receptors, including the folate receptor and CD44, exhibit potential in the targeted delivery of therapeutic agents that not only disrupt epithelial‒mesenchymal transition (EMT) but also attenuate metastatic dissemination in TNBC [66].

Recent investigations have elucidated the contributory function of insulin resistance in the amplification of triple-negative breast cancer (TNBC) aggressiveness via exosome-mediated epithelial‒mesenchymal transition (EMT) induction. Exosomes derived from insulin-resistant adipocytes have been shown to upregulate pro-EMT gene expression and augment metastatic phenotypes in both in vitro and in vivo experimental frameworks, establishing a correlation between metabolic dysregulation and unfavorable clinical outcomes in breast cancer patients [67]. This multifaceted modulation by exosomes integrates intricate networks of signaling pathways, activation of transcription factors, and remodeling of the extracellular matrix, ultimately orchestrating the metastatic process in breast cancer and TNBC [68].

## Advanced chromatographic techniques for exosome characterization

Exosomes are membrane-bound vesicles secreted by most cell types and carry a range of cargo proteins, lipids, nucleic acids and metabolites, reflecting the state and identity of their parent cells [69]. Their potential as noninvasive biomarkers for early diagnosis and their utility in therapeutic drug delivery have driven significant research into their isolation and characterization [70]. Traditional isolation methods such as ultracentrifugation, while extensively used, suffer from limitations related to yield, purity, and potential damage to exosomal structures [71]. The implementation of advanced chromatographic methodologies has escalated to overcome the prevailing limitations by facilitating mild yet effective separation mechanisms predicated upon size, surface affinity, or an amalgamation of physicochemical characteristics (Table 2) [82].

**Table 2:** Comparative performance of major exosome isolation techniques, including ultracentrifugation (UC), size exclusion chromatography (SEC), immunoaffinity chromatography (IAC), and high-performance liquid chromatography (HPLC), with respect to yield, purity, time efficiency, and cost or equipment considerations

Method	Yield	Purity	Time efficiency	Equipment/cost factors	Ref
Chromatographic methods					
Thin-layer chromatography (TLC)	High	Moderate	Fast	Low	[72]
Column chromatography	Moderate	High	Moderate	Moderate	[73]
Size exclusion chromatography (SEC)	Moderate	Moderate	Moderate	Moderate	[74, 75]
Hydrophobic interaction chromatography (HIC)	High	High	Moderate	Moderate	[76]
Anion exchange chromatography (AIEX)	Moderate	High	Moderate	Moderate	[77]
Non-chromatographic methods					
Ultracentrifugation (UC)	Moderate	Low	Slow	High	[74, 78]
Precipitation	High	Low	Fast	Low	[74, 79]
Ultrafiltration	Moderate	Moderate	Fast	Moderate	[79]
Immunoaffinity	Moderate	High	Moderate	High	[80]
Microfluidics	Moderate	High	Fast	High	[81]

### Size-based chromatographic techniques

#### Size exclusion chromatography

Size exclusion chromatography (SEC) is based on molecular sieving, in which a stationary phase composed of a porous gel matrix (such as Sepharose or dextran-based resins) allows for the separation of particles on the basis of hydrodynamic size [71]. In this strategy, large particles, i.e., exosomes, cannot pass through the pores of a gel to elute quickly, whereas smaller contaminants are preferentially entrapped in resin pores and can elute later; hence, the enriched fraction of exosomes that retain purity and biological activity is enhanced [71]. SEC has been commercialized in several formats, including kits such as qEV and ExoEasy, which have gained popularity because of their ease-of-use reproducibility as well as their applicability to clinical sample volumes [83]. By adopting gentle SEC separation conditions, minimal chemical and high shear will be achieved, minimizing the risk of exosome aggregation or deformation and preserving the integrity of their biophysical and functional material during isolation [84]. Additionally, improvements in methodology (e.g., SEC with ultrafiltration (SEC-UF)) have greatly increased yield and purity for the isolation of intact vesicles suitable for downstream microscopy, nanoparticle tracking analysis (NTA), and mass spectrometric characterization [84].

**Fig. 4:**
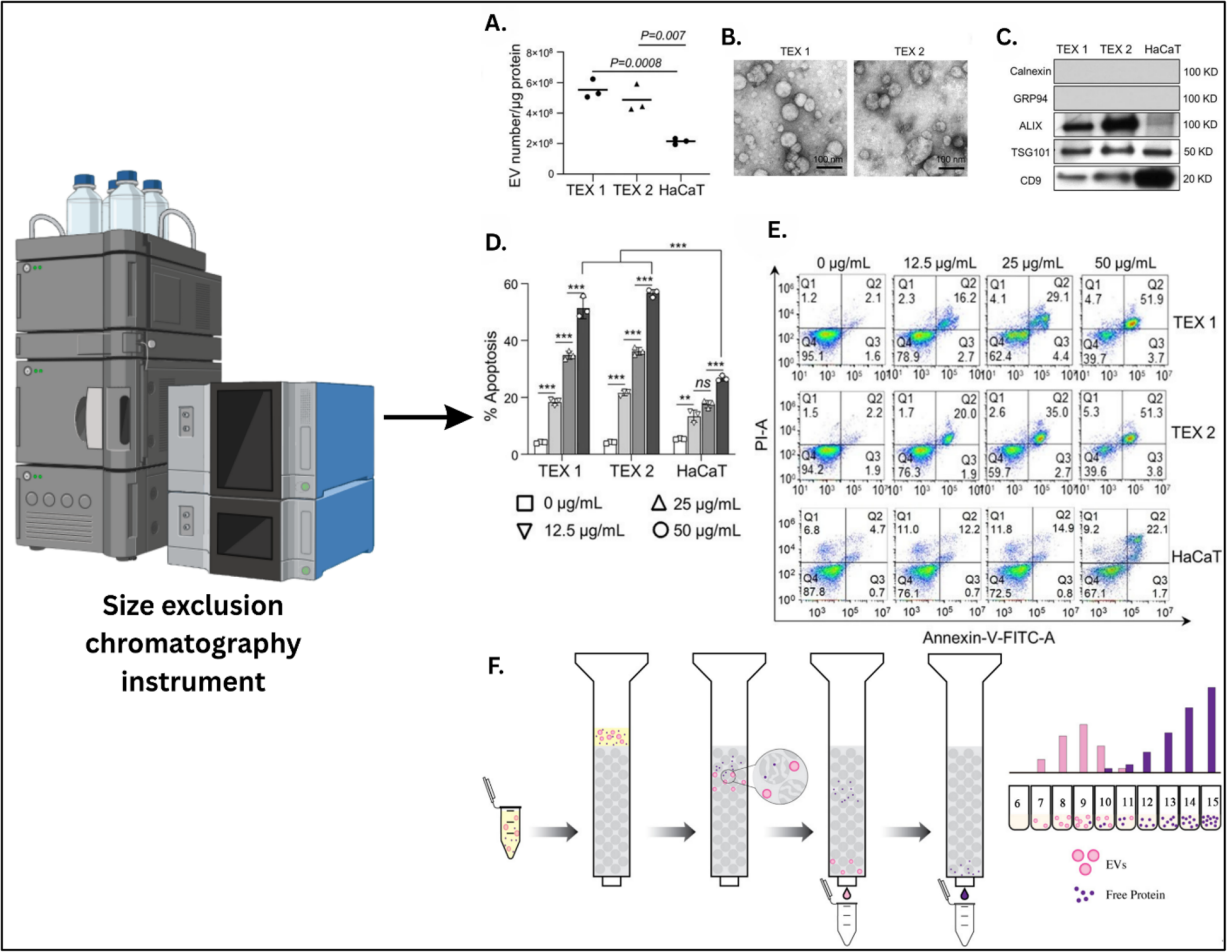
Application of size exclusion chromatography (SEC) in the isolation and functional characterization of tumor-derived small extracellular vesicles (sEVs) in triple-negative breast cancer (TNBC). **A** Quantitative analysis of the vesicle concentration (EV number/µg protein) in SEC fraction #4, which revealed significantly greater EV yields in TEX1 (MDA-MB-231) and TEX2 (MDA-MB-436) than in nonmalignant HaCaT-derived vesicles. **B** Transmission electron microscopy (TEM) images of TEX1 and TEX2 confirm the sEV morphology and size distribution (~ 100 nm). **C** Western blot validation of canonical exosomal markers (CD9, ALIX, and TSG101) and the absence of cellular contaminants (calnexin and GRP94), confirming the purity of the SEC-isolated vesicles. **D** Quantitative analysis of CD8⁺ Jurkat T-apoptosis following exposure to increasing concentrations of TEX1, TEX2, or HaCaT sEVs, demonstrating the dose-dependent immune modulatory effects of TNBC-derived vesicles. **E** Representative flow cytometry plots showing Annexin-V and PI staining of apoptotic Jurkat cells after treatment with 0–50 µg/mL EVs (adapted from Mondal, Kumar, et al., under the terms and conditions of the Creative Commons Attribution (CC-BY) license (CC-BY 4.0)). **A**–**E** Available at [85]. **F** Schematic diagram of the SEC workflow: larger vesicles elute in early fractions, whereas smaller proteins are retained longer, allowing efficient separation of extracellular vesicles (EVs, pink) from soluble protein contaminants (purple). The process preserves EV integrity and is ideal for downstream functional or omics analyses (adapted from Shah et al. under the terms and conditions of the Creative Commons Attribution (CC-BY) license (CC-BY 4.0)). **F** Available at [86]

The application of SECs is well exemplified in Fig. 4. As shown in subfigure (A), quantitative analysis of the vesicle concentration (EV number/µg protein) in SEC fraction 4 revealed that the malignant TNBC cell lines TEX1 (MDA-MB-231) and TEX2 (MDA-MB-436) yielded significantly greater amounts of EVs than vesicles derived from nonmalignant HaCaT cells [85]. This observation underscores the ability of the SEC to capture tumor-specific vesicle secretion, which is a critical parameter when investigating disease biomarkers and functional heterogeneity in TNBC. The morphological integrity and size distribution of the isolated vesicles are crucial for downstream functional assays. As shown in Fig. 4 (B), transmission electron microscopy (TEM) images of the TEX1 and TEX2 preparations confirmed the presence of sEVs with a typical cup-shaped morphology and size distribution of approximately 100 nm. Preservation of these biophysical properties is essential because alterations in vesicle structure could compromise not only cargo delivery but also the biological response elicited by recipient cells. Moreover, the purity of the SEC‐isolated vesicles was rigorously validated by Western blot analysis, as presented in Fig. 4(C). The expression of canonical exosomal markers such as CD9, ALIX, and TSG101 confirms successful enrichment of vesicles, whereas the absence of cellular contaminants such as calnexin and GRP94 further attests to the high purity achieved with SEC. This level of purity is pivotal for downstream applications, ensuring that the observed biological effects can be directly attributed to sEVs rather than to confounding coisolated proteins or debris. The functional impact of TNBC‐derived sEVs on immune cells is shown in Fig. 4(D) and Fig. 4(E). Quantitative analysis revealed dose‐dependent induction of apoptosis in CD8⁺ Jurkat T cells upon treatment with TEX1 and TEX2 vesicles. In contrast, sEVs from nonmalignant HaCaT cells produce minimal immunomodulatory effects. These results highlight the immune regulatory capacity of tumor-derived vesicles, implicating them in the modulation of antitumor immune responses—a factor critical for understanding TNBC progression and therapeutic resistance [85]. Finally, Fig. 4(F) presents a schematic diagram of the SEC workflow. The diagram shows that larger vesicles elute in early fractions, whereas smaller soluble proteins are retained longer. This separation ensures efficient isolation of extracellular vesicles with preserved integrity, making SEC particularly well suited for subsequent functional analyses and omics studies [86].

#### High-performance liquid chromatography

Recent advances in chromatographic techniques, particularly high‐performance liquid chromatography (HPLC) variants, have emerged as transformative tools for isolating and characterizing exosomes derived from three‐dimensional (3D) culture models of triple‐negative breast cancer (TNBC), offering enhanced resolution in the separation of highly heterogeneous exosome subpopulations that more accurately recapitulate the in vivo tumor microenvironment (Clinical Trial Search: Chromatography exosome analysis AND triple-negative breast cancer AND 3D culture). These chromatography‐based methods, which include size‐exclusion and affinity‐based methods and are exemplified by particle purification liquid chromatography (PPLC), address longstanding challenges inherent in conventional ultracentrifugation, such as vesicle aggregation and co‐isolation of contaminating plasma proteins and lipoproteins, thereby yielding purer exosome fractions for downstream functional analysis [87]. When engineered into 3D culture systems (e.g., organoids, spheroids, and scaffold-modeled HPLC variants that best represent cell‒cell and cell‒matrix interactions), exosomes with more faithful cargo, such as proteins, more precisely reflect the aggressive phenotypes observed in TNB [88]. High-resolution chromatographic separations combined with mass spectrometry and other omics‐based analytical platforms provide greater power in profiling complex exosomal biomolecules to elucidate the molecular basis for TNBC progression, chemoresistance, and metastatic potential [89]. With this method, we can discover unique exosomal markers that predict how individuals respond to treatments and conduct multi‐omics analyses for biomarker research and new targeted drugs. In addition, when HPLC is combined with novel 3D culture models, researchers can achieve a greater degree of specificity and purity in exosome isolation, allowing functional assays to be robust and thus able to explain the different roles of various exosome subpopulations in the modulation of the TME. Compared with the conventional two-dimensional culture system, the combination of advanced chromatographic methods with 3D cultures opens new avenues for exploring exosome heterogeneity in TNBC using a setup that not only amplifies throughput but also maintains the bioactivities of the exosomes present, consequently initiating clinical translational investigations without the support of the present lack of registered clinical studies synthesizing the use of these techniques [88, 90]. The combination of 3D culture models and high-performance liquid chromatography (HPLC)‐based chromatographic methods represents a promising path forward for identifying the productive molecular complex and functional shakes of TNBC exosomes, which could be further translated into novel biomarkers and the development of targeted therapeutic interventions [91].

### Affinity-based chromatographic techniques

#### Immunoaffinity chromatography

The combination of immunoaffinity chromatography (IAC) with advanced chromatographic techniques and three-dimensional (3D) culture models is a very interesting initiative to decode the heterogeneity and purpose of exosomes in triple-negative breast cancer (TNBC). With the help of immunoaffinity chromatography, the exosome subpopulations are targeted by antibodies to capture specific protein markers on the vesicle surface; thus, one can obtain the homogeneous vesicle fractions necessary for making correct downstream functional evaluations [92]. This highly selective capture method, together with more advanced chromatography techniques such as high-performance liquid chromatography (HPLC), size exclusion chromatography, and ion-exchange chromatography, enables the discrimination of exosome subtypes on the basis of their biophysical properties and molecular cargo. These combined methods effectively help separate exosomes from interfering proteins and other extracellular vesicles, thus preserving their bioactivity and increasing the reproducibility of proteomic and transcriptomic analyses [93]. Furthermore, when 3D culture models with more cell–cell interactions, extracellular matrix dynamics, and hypoxic gradients that more accurately mimic the in vivo tumor microenvironment are incorporated, the generated exosomes closely resemble those in patient tumors [88]. In this physiologically relevant context, researchers can elucidate the specific exosomal surface markers and cargo distribution that are primarily responsible for TNBC tumor progression, metastasis, and chemoresistance phenotypes owing to the combination of immunoaffinity capture and advanced chromatographic separation techniques [94]. Moreover, the use of such combined systems leads not only to increased purity and yield when exosomes from 3D cultures are isolated but also to the use of high-resolution profiling approaches that are capable of finding new biomarkers and therapeutic targets. In this way, the strategy presented in this publication overcomes the problem of a transition from basic to applied research through the creation of powerful methods for functional and complex exosome analysis in solid cancer and, finally, advances in the application of targeted drugs and personalized therapy in triple-negative breast cancer [95].

### Hyphenated techniques and integration with mass spectrometry

A critical advancement in exosome characterization is the integration of chromatographic separation with mass spectrometry (MS), an approach commonly referred to as liquid chromatography‒mass spectrometry (LC‒MS), or high-performance liquid chromatography‒mass spectrometry (HPLC‒MS) [96]. Liquid chromatography is applied to the isolation of exosome proteins, lipids, and metabolites from exosomes before their subsequent mass spectrometric deciphering, which enables thorough and sensitive profiling of the cargo carried [71]. The adoption of UHPLC‒MS/MS in proteomics has produced a powerful platform for the mass analysis of exosomes, which confirms its ability to detect thousands of protein molecules in exosome samples. This provides the possibility of identifying swarms of disease-specific biomarkers and determining the functions and forms of exosomes [1]. Multidimensional chromatography approaches, such as the sequential use of strong cation exchange (SCX) followed by reversed-phase (RP) chromatography, have further increased the resolution and separation power, allowing for in-depth proteomic profiling and the detection of posttranslational modifications crucial to exosome biology [71].

### Standardization issues in exosome isolation and clinical translation

Standardization issues in exosome isolation and clinical translation remain major obstacles to both scientific progress and the reliable application of exosome-based therapeutics. Current isolation methods, including ultracentrifugation, size-exclusion chromatography, precipitation kits, and immunoaffinity approaches, differ significantly in terms of yield, purity, scalability, cost, and reproducibility. This methodological heterogeneity leads to considerable batch-to-batch variability in both the quantity and quality of exosomes, impacting the consistency of experimental outcomes and therapeutic interventions [97].

Contamination with extracellular vesicles (EVs), proteins, and other nonexosomal materials frequently occurs, especially in precipitation-based techniques, diminishing the functional purity and biological relevance of isolated exosome populations. Furthermore, the absence of universally accepted markers and standardized protocols for exosome identification and characterization complicates reliable profiling, causing variations not only between laboratories but also between batches within a single laboratory [98].

For clinical translation, issues of scalability and process validation are even more pronounced. Large-scale, GMP-compliant, and reproducible production protocols for exosomes are currently lacking, which makes it challenging to meet the stringent requirements for clinical-grade therapeutics [99]. Additionally, regulatory frameworks for exosome therapies are still evolving, and the field lacks consensus on key criteria such as potency, release testing, and long-term safety assessment.

Despite international efforts such as the incrementally updated MISEV guidelines from the International Society for Extracellular Vesicles and data-sharing initiatives such as EV-TRACK, significant challenges in transparency, methodological rigor, and cross-study comparability remain. The inconsistency and heterogeneity of exosome preparations have direct consequences for their efficacy and safety in human applications, ultimately slowing their clinical adoption and regulatory approval [100].

Advancements in isolation technologies (such as microfluidic and label-free strategies), implementation of detailed reporting guidelines, and interdisciplinary collaborations are promising approaches under exploration to address these limitations. However, until robust standardization is achieved across isolation, characterization, and clinical application, the reproducibility and translational impact of exosome research will remain constrained [101].

## Advances in 3D culture models for TNBC

Breast cancer is a major concern in many countries, as it is the most common cancer among women and because it causes many diagnoses and deaths. In the field of oncology, identifying new breast cancer treatments is a top priority [102]. The ongoing search for innovative breast cancer treatments is a critical focus in oncology research. However, many therapies that show promise in laboratory (in vitro) studies often fail to deliver similar results in clinical settings. This discrepancy is largely attributed to the reliance on 2D cell culture models, which do not accurately replicate the complex biological environment of tumors found in the human body [103, 104]. The evidence suggests that 3D models are able to more accurately show how tumors function. Experiments indicate that spheroids cause fewer cancerous breast cells to proliferate and become more drug resistant than the same type of cells grown in 2D culture. 3D models also reveal how cells interact, including E-cadherin, which forms junctions, and other factors that are Linked to drug resistance and the ability to spread locally or elsewhere. In addition, tests with chemical drugs have shown that 3D-generated tumors have greater resistance, which is most likely linked to stronger interactions with microenvironment cells. The ATR-Chk1 signaling pathway has been shown to break through the resistance found in cells grown in 3D [105]. Garg and Hunt explored in a recent study how breast cancer cells react to chemotherapy in several types of culture systems, such as 3D-printed systems. It was discovered that growing cancer cells together with endothelial cells in 3D tissue made it easier to determine how the drugs worked, reflecting how 3D better represents the body [106]. Another study by Breslin and O’Driscoll (2016) reported that 3D cultured breast cancer cells were more resistant to drugs and had different levels of important survival proteins and enzymes than 2D cells. Researchers have guided the use of 3D models for drug testing [107]. Yousafzai and associates (2024) examined how 3D models can be used to study exosome-related signaling pathways in triple-negative breast cancer. 3D culture increases the effectiveness of exosomes and increases the accuracy of drug delivery, which is crucial for targeted therapy [108].

### Biological rationale for 3D culture models

#### Recapitulation of the in vivo architecture

A main benefit of 3D culture systems is their ability to reflect how breast tumors are structured in the body. Under 3D culture conditions, MCF-7, BT-549, T-47D, and MDA-MB-231 breast cancer cells organize themselves into spheroids with lumens and have the ability to invade the space around them, similar to real patient tumors. Studies have shown that the shapes of cells in 3D culture vary greatly, such as the compact spheroids typical of MCF-7 colonies and the loose invasive growth of triple-negative breast cancer models, which mirrors the variety seen in actual tumors [109]. These morphological features are critical for understanding invasion, metastasis, and localized drug resistance, as the physical organization of cells influences both cell signaling pathways and the diffusion of therapeutic agents.

#### Impact of the extracellular matrix and mechanical cues

3D systems not only emphasize the cellular architecture but also successfully recapitulate the extracellular matrix (ECM) environment. The ECM in tumors provides both chemical signals and mechanical properties, such as stiffness and softness, to influence how cells behave. For example, cancer cells from breast tumors exhibit malignant features when grown in a mixture of alginate and Matrigel, which mimics the mechanical conditions of invasive ductal carcinoma [110]. These studies underscore that those mechanical properties (e.g., stiffness measured via nanoindentation techniques) directly influence cell adhesion, proliferation, and invasion. This improved recapitulation of in vivo mechanical properties is essential for studies investigating the response of cancer cells to chemotherapeutic agents, as ECM-induced mechanical signals can alter intracellular signaling pathways, including the AKT-mTOR-S6K cascade [109].

#### Hypoxia and gradients of bioactive molecules

Hypoxia and concentration gradients of bioactive chemicals in the tumor environment are known to promote tumor growth and spread the development of treatment resistance. In three-dimensional (3D) cultures, oxygen, nutrients, and signals are restricted in diffusion, which leads to gradients; this makes 3D conditions more appropriate than usual two-dimensional (2D) cultures for exploring tumor functions [109, 111]. The presence of such gradients enhances the physiological relevance of 3D culture models in breast cancer research, enabling researchers to study the emergence of dormant cell populations and cancer stem-like cells that contribute to therapy resistance.

In traditional 2D culture, cells always receive the same external factors, whereas real tumors exhibit very different oxygen and nutrient levels depending on where they are inside the body. In contrast, 3D spheroids and engineered scaffold cultures provide spatial gradients that are more closely related than they are in in vivo stages, such as the formation of low-oxygen regions at the center of the spheroid, which causes various chemical and cellular changes [111]. These gradients are not limited to oxygen but extend to bioactive molecules such as metabolites, lipids, cytokines, and growth factors that collectively shape the metabolic and signaling landscape of tumors [109]. Microfluidics, 3D bioprinting, and scaffold engineering developments give researchers the ability to control these gradients in the laboratory. Microfluidics allows the formation of gradients for both oxygen and nutrients in 3D hydrogels, making it possible to observe what happens to cancer cells as a result. Importantly, the level of control is high since the location of hypoxia inside a tumor can change both cellular metabolism and important changes in cell traits, such as EMT, which helps tumors become metastatic and harder to treat with drugs [111].

Overall, the integration of hypoxic gradients and gradients of bioactive molecules in 3D breast cancer culture systems is a critical aspect of tumor modeling that advances our understanding of cancer biology while offering new avenues for therapeutic intervention. Continued multidisciplinary research and technological innovations in this area are expected to further elucidate the mechanisms by which hypoxia and environmental gradients drive breast cancer progression and treatment resistance, thereby enabling the development of more effective, targeted strategies for combating this complex disease.

### Tumor spheroids and organoids

The use of tumor spheroids and patient-derived organoids (PDOs) has greatly advanced research on triple-negative breast cancer, mainly to explore the role of exosomes in causing tumors to spread, become resistant to drugs, and metastasize. In contrast to ordinary 2D cultures, these models are designed to recapitulate the conditions inside tumors. They accomplish this by saving cell‒cell connections, the ECM, and stromal cells, which leads to data that more accurately fit clinical findings in real patients with TNBC [112]. Exosomes, nanosized vesicles secreted by cancer and stromal cells, play pivotal roles in intercellular communication, modulating pathways such as angiogenesis, epithelial‒mesenchymal transition (EMT), and immune evasion [88]. In 3D models, exosomes become more efficient, and their cargo (including proteins and noncoding RNAs) adjusts to fit the hypoxic and nutrient-poor reality of solid tumor tissues. For example, spheroids grown in 3D culture release more exosomal translesion DNA polymerases and parts of the ATR-Chk1 pathway, which help TNBC cancer cells resist cisplatin by repairing DNA and allowing the cells to progress through the cycle [113]. These findings underscore how 3D models capture exosome-mediated adaptive responses to chemotherapy.

**Fig. 5:**
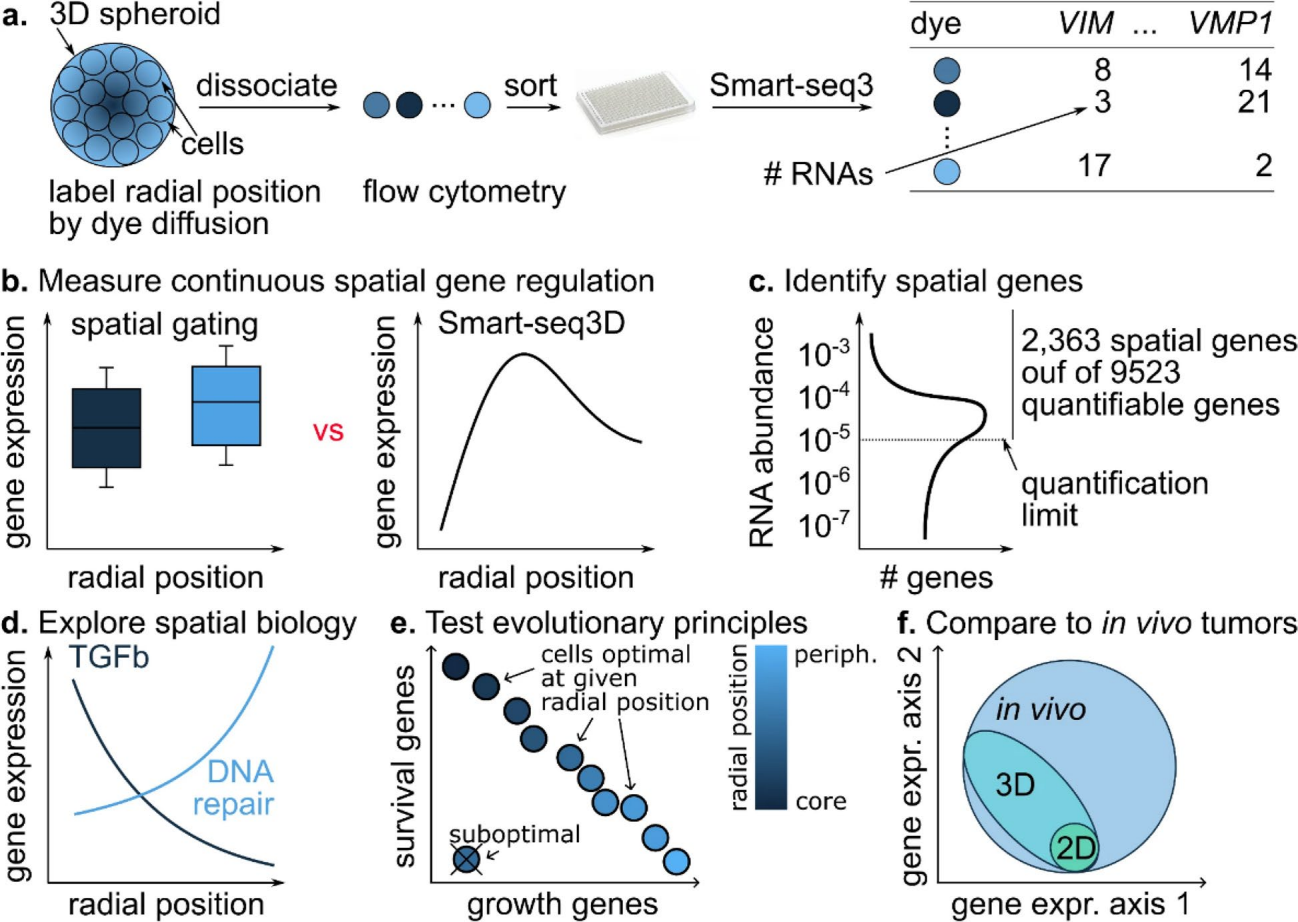
**a** Smart-seq3D integrates dye diffusion, Smart-seq3xpress, and probabilistic inference to map single-cell transcriptomes in tumor spheroids. **b** Smart-seq3D reveals ungated, continuous gene expression patterns. **c** Deep single-cell transcriptomics via Smart-seq3 revealed 2363 spatial Genes among 9523 genes with an average of at least one RNA copy per cell. Applying Smart-seq3D to tumor spheroids enabled **d** exploration of spatial tumor biology, **e** testing evolutionary principles of tumor heterogeneity, and **f** revealing aspects of the transcriptional heterogeneity of in vivo tumors captured by 3D spheroids (adapted from Cougnoux et al. under the terms and conditions of the Creative Commons Attribution (CC-BY) license (CC-BY 4.0)). Available at [113]

Furthermore, the study introduces Smart-seq3D, a tool that unites dye diffusion, Smart-seq3xpress (for high-coverage transcriptome sequencing of single cells) and a statistical inference approach to perform spatially resolved, single-cell transcriptomics in tumor spheroids. The application of Smart-seq3D to triple-negative breast tumor spheroids provides better spatial information and more detailed gene profiles, which can help identify thousands of regulated genes with different patterns, even some that do not appear in profiles from two-bin sort-and-sequence techniques. This method allows the study of cellular functions in individual spheroid regions by examining pathways that are tested and confirmed by staining and drugs. In addition, because Smart-seq3D allows researchers to examine Living tissue and organs, it is possible to analyze evolutionary aspects of spatial heterogeneity and discover intraspheroid heterogeneity present only in tumors grown in 3D but not in 2D cultures (Fig. 5) [113].

Using 3D models together with multiomics data has revealed possible targets for overcoming TNBC resistance. Research using coculture models revealed that exosomes made by stromal cells play a role in shielding cancer cells from T-cell activity by sending immunosuppressive signals (such as PD-L1) to them. Using engineered exosomes with ATR- or MEK-targeted drugs could help reverse resistance to chemotherapy in various tests [112]. These insights, supported by the comparative features outlined in Table 3, highlight how tumor spheroids and organoids bridge the gap between in vitro and in vivo systems, offering a powerful platform to study exosome dynamics and drive therapeutic innovation in TNBC.

**Table 3:** Comparison of tumor spheroids/organoids and 2D cultures across key experimental features

Feature	Tumor spheroids & organoids	2D cultures	Ref
Oxygen & nutrient gradients	Create physiologically relevant gradients, with hypoxic cores and nutrient-depleted zones	Uniform oxygen and nutrient exposure	[114, 115]
Hypoxia modeling	Naturally, develop hypoxic cores useful for testing hypoxia-specific drugs	Limited or artificial hypoxia induction	[116]
Proliferation gradient	Distinct zones of proliferation, quiescence, and necrosis replicate real tumor architecture	Homogeneous proliferation	[117]
Drug resistance	Spheroids show higher drug resistance due to limited diffusion and hypoxic conditions	Often overestimates drug efficacy due to ideal conditions	[118, 119]
Cell heterogeneity & microenvironment	Maintain tumor heterogeneity, stromal interactions, and ECM properties	Poor representation of tumor microenvironment	[120, 121]
Drug screening & translational relevance	More predictive for in vivo efficacy and patient response	Lower predictive accuracy for clinical outcomes	[122, 123]

By bridging the gap between in vitro and in vivo biology, tumor spheroids and organoids provide a robust platform for unraveling exosome heterogeneity and accelerating therapeutic innovation in TNBC.

### 3D bioprinting and hydrogel scaffolds

#### 3D bioprinting in TNBC

3D bioprinting has emerged as a transformative approach for modeling triple-negative breast cancer (TNBC), particularly for replicating its aggressive tumor microenvironment (TME) and cancer stem cell (CSC) dynamics. Recent advancements have focused on creating biomimetic platforms to study therapy resistance and improve drug screening [124]. 3D bioprinting has emerged as a transformative platform for constructing advanced in vitro cancer models, enabling the fabrication of tissue-mimetic structures with high spatial precision and biological complexity. According to Sztankovics et al. [125], triple-negative breast cancer (TNBC) benefits from the use of biofabricated models because its unique characteristics make it easier to study in more realistic microenvironments than traditional cultures or spheroids. By using alginate–gelatin bioinks and an extrusion-based printer, researchers created TNBC models, including the MDA-MB-231 and MDA-MB-468 cell Lines, which showed good cell survival, continued cell proliferation for several days, and the formation of tissue-like tissues. These 3D models better represent how tumors differ in metabolism and look because various genes, such as ALDH1, COXIV, and LDHA, are expressed differently, as they are expressed in real tumors. Importantly, these bioprinted structures resemble xenografts in response to treatments with doxorubicin and rapamycin, stressing their value for preclinical experiments. Moreover, integrating stromal components such as endothelial cells and adipocytes into bioprinted TNBC models enhances their physiological relevance by mimicking tumor-stroma interactions, angiogenesis, and the immune landscape [126].

Despite the important achievements, variability in mixes, inconsistent methods from research to research, and technical problems are still present. In addition, as bioink formulas improve, more crosslinking options are applied, and the printing resolution increases. 3D bioprinting will soon offer well-made, repeatable, and individualized TNBC models for both study and testing.

Another study by Sztankovics et al. [125] investigated the creation of a 3D bioprinted tissue model for breast cancer using the MDA-MB-231 and MDA-MB-468 triple-negative cell Lines. Using alginate-gelatin hydrogels, these bioprinted models add tumor cells, permitting a closer simulation of real tissues than are used in 2D cultures. 3D bioprinted structures Look more complex in terms of their shape and cell distribution, much Like real tumors found in the body. Additionally, Alamar blue and SRB assays indicated that the tissue remained viable and grew throughout 21 days, accompanied by a unique distribution of biomarkers involved in metabolism and cell death (COXIV, LDHA, ALDH1, and cleaved caspase-3) along the structure. These 3D bioprinted models closely reflect how native TNBC tumors are affected by metabolism and resistance to drugs, which is useful for preclinical drug evaluations. Sztankovics et al. highlighted that bioprinted models help connect the gap between data from Petri dishes and results in laboratory animals, which is especially useful for predicting which drugs may best help TNBC patients.

3D bioprinting and printing technologies are now important tools for handling personalized treatment for the inflammatory type of triple-negative breast cancer (TNBC). Recently, Huang et al. [127] described how a 3D-printed tumor replica derived from PET/CT information was applied to triple-negative breast cancer (stage IIIC). A model was built via desktop vat polymerization, including details of the tumor, skin, and muscles of the chest from the patient. The 3D representation helped the patient feel and visualize the extent of the disease, so the conversation about treatment plans became much easier. The printing process involved segmenting CT imaging data, referencing PET scans for tumor delineation, and fabricating multiple components via different resins to represent anatomical structures. The resulting Life-sized model was instrumental during consultations, enabling clearer visualization of the tumor-to-breast ratios and spatial relationships critical for surgical planning. Additionally, the model supported multidisciplinary medical education and enhanced communication among radiologists, surgeons, and oncologists involved in patient care. This is an example of how 3D printing could improve how doctors prepare for surgery and can also encourage patients and their caregivers to join in decision-making. Because desktop 3D printing is becoming more widely available, the use of patient-specific bioprinted models in medical oncology is likely to increase.

3D bioprinting has proven useful because it allows the construction of tissues that are similar to real breast cancer samples with both a detailed design and accurate biology. Dankó and his team [128] created a model by printing Human breast cancer cells in an alginate-gelatin Gel to evaluate whether this model is reliable and provides results similar to those of 2D, 3D spheroid, and xenograft models. Bioprinted tissue-mimicking scaffolds (TMSs) support the growth of tumor tissue and form channels, with their cell structures and metabolic activity matching those of xenografted tumors, as indicated mainly by the presence of the p-S6, FASN, p-ACC, and LDHA proteins. Compared with conventional cultures, the 3D bioprinted model produced more accurate drug sensitivity profiles, particularly with respect to in vivo resistance to rapamycin, doxycycline, and doxorubicin, which was not observed in 2D or spheroid systems. Combination treatments with rapamycin and doxycycline demonstrated enhanced efficacy in both the TMS and xenograft models, highlighting the relevance of this platform for preclinical screening. Moreover, immunohistochemistry revealed metabolic enzyme expression heterogeneity in TMSs closely matching xenograft patterns, with Shannon diversity index (SDI) scores supporting the reproducibility of in vivo-like metabolic plasticity. The study concludes that bioprinted TMSs offer a superior in vitro alternative to traditional models for breast cancer drug testing, bridging the gap between preclinical assays and in vivo behavior. These findings underscore the importance of integrating 3D bioprinting into cancer research pipelines to reduce reliance on animal models while enhancing physiological accuracy.

### Microfluidic tumor-on-chip platforms

Microfluidic tumor-on-chip platforms have emerged as transformative tools for modeling cancer biology and testing therapeutics in vitro with unprecedented physiological relevance [129]. These platforms leverage microengineering techniques to recreate the tumor microenvironment (TME) within a microscale device, enabling precise spatial and temporal control over factors such as fluid flow, oxygen gradients, and cellular architecture [130]. Usually, tumor-on-chip systems contain microchannels made of biocompatible materials such as polydimethylsiloxane (PDMS), and cells/structures such as cancer cells, stromal cells, endothelial cells, and the extracellular matrix (ECM) are placed inside [131]. Similar to Human cells in a tumor, these systems can demonstrate angiogenesis, with cells from the immune system moving in and expressing metastasis, making them better models than regular 2D cultures do [132].

One of the key advantages of microfluidic tumor models is their capacity to simulate interstitial fluid pressure and dynamic perfusion conditions, mimicking in vivo tumor physiology [129]. This allows researchers to study drug penetration, cellular responses to shear stress, and the effects of nutrient and oxygen gradients on tumor behavior. Furthermore, real-time imaging and sampling capabilities make these platforms highly adaptable for high-throughput screening and longitudinal studies [133]. Combining elements of biosensors and organ-specific environments now allows the coculture of tumor tissue with organoids or healthy tissue from other organs (for example, liver-on-a-chip) to assess the harmful impact on the body and the risk of metastasis. Additionally, tumor cells derived from patients are often used to create personalized tumor-on-a-chip systems, supporting new applications in precision oncology [134].

Despite their promise, challenges remain, including standardization of device fabrication, scalability for clinical translation, and integration of immune components for immunotherapy testing [135]. Ongoing interdisciplinary efforts in materials science, cell biology, and bioengineering are expected to address these limitations and further enhance the fidelity and utility of tumor-on-chip platforms in cancer research and drug development [136].

**Fig. 6:**
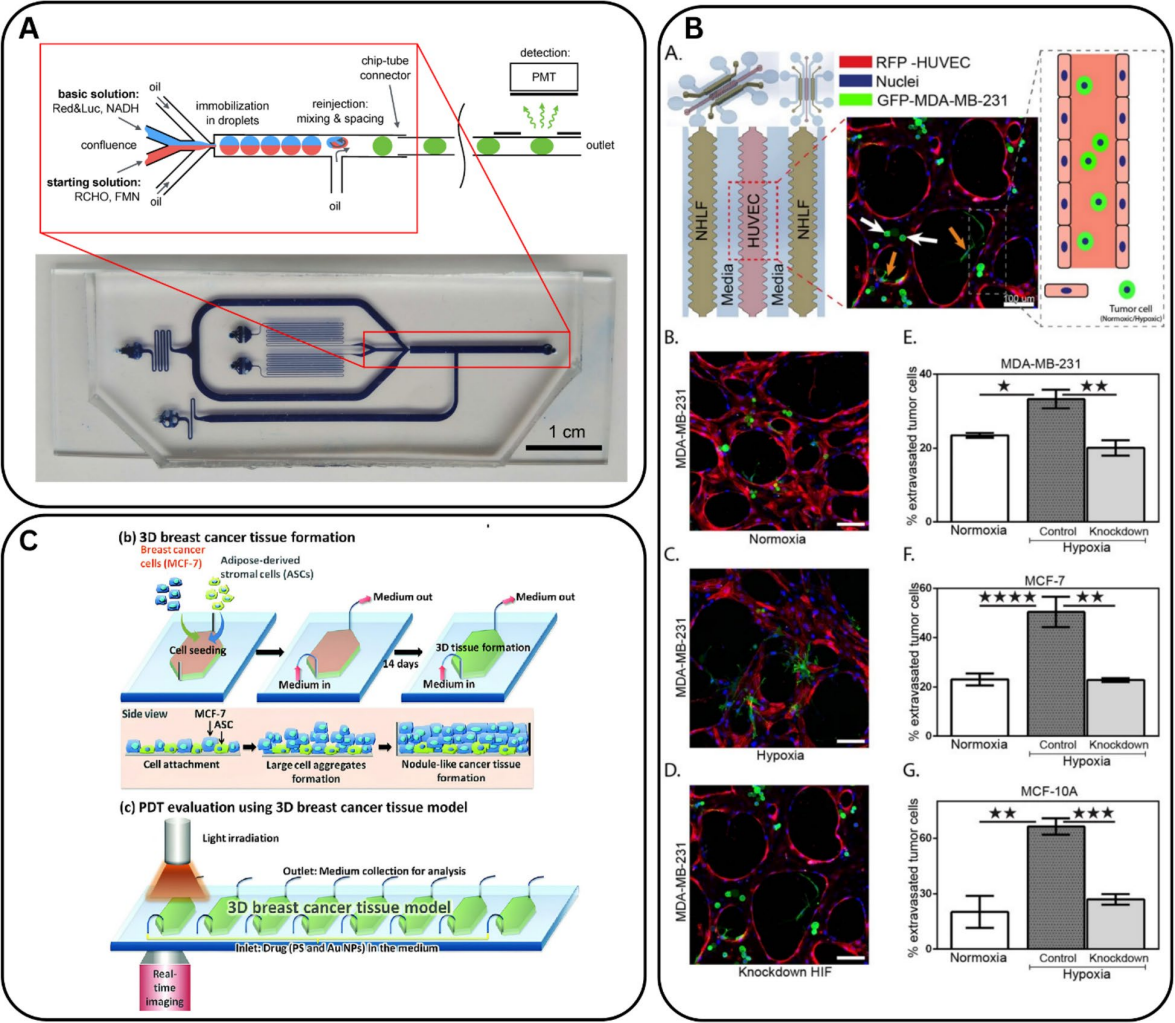
**A** Schematic and photograph of the microfluidic chip used for droplet-based encapsulation and detection assays. The top panel shows the layout of droplet immobilization, mixing, spacing, and fluorescence detection via PMT. The bottom panel presents the actual device with dyed channels: scale bar = 1 cm (adapted from Yakimov, Anton S., et al. under the terms and conditions of the Creative Commons Attribution (CC-BY) license (CC-BY 4.0)) available at [138]. **B** In vitro 3D microvascular extravasation model. **A** Schematic of the three-gel channel chip: RFP-labeled HUVECs are seeded in the central gel channel to form the vasculature; adjacent channels contain NHLFs for structural support. GFP-labeled MDA-MB-231 tumor cells were used to study transendothelial migration. A representative fluorescence image shows vascular structures and tumor cells; orange arrows denote extravasated cells, and white arrows denote nonextravasated cells. **B**–**D** Fluorescence images of MDA-MB-231 cells under (**B**) normoxic, (**C**) hypoxic, and (D) HIF-1α-knockdown conditions. **E**–**G** Quantification of extravasated MDA-MB-231 (E), MCF-7 (F), and MCF-10A (**G**) cells under these conditions. Scale bar = 100 µm; mean ± SEM, *p < 0.05, **p < 0.01, ***p < 0.001, *n* = 5 (adapted from Song, Jiho, et al. under the terms and conditions of the Creative Commons Attribution (CC-BY) license (CC-BY 4.0)) available at [139]. **C** (b) Schematic of 3D breast cancer tissue formation via coculture of MCF-7 breast cancer cells and adipose-derived stromal cells (ASCs) over 14 days, resulting in nodular-like tumor tissue. (c) Photodynamic therapy (PDT) evaluation setup using the developed tissue model: drugs (PS and AuNPs) are introduced via an inlet, and light is applied from above for treatment assessment with real-time imaging and outlet sampling (adapted from Mohammad-Hadi et al. under the terms and conditions of the Creative Commons Attribution (CC-BY) license (CC-BY 4.0)) available at [137]

Microfluidic tumor-on-chip platforms are now commonly used to recreate the structure and function of the tumor microenvironment within the laboratory. Using microscale physics, they can replicate things such as interstitial flow, gradients of nutrients, and mechanical factors within the three-dimensional model of a tumor. An important example is the invention of droplet-based microfluidic chips, which make it possible to organize reactions and track what is happening in biological experiments. The chips allow for the precise production and flow of nanoliter droplets, which work as single bioreactors and make it possible to study tumors by analyzing reactions to different treatments or factors. Luciferase-based optical biosensing adds extra value because it allows for real-time and safe detection of changes in biochemical measures triggered by toxins or therapies. In their study, Hadi et al. [137] made a device called a droplet microfluidic chip to produce small droplets containing bacterial luciferase and cofactors, which enabled them to detect copper ions, a model pollutant, very sensitively. Figure 6 (A) (obtained from the original paper) shows the layout of the chip, its flow control, and the method of luminescence detection with photomultiplier tubes. With this technology, we can see that tumor-on-chip systems have uses both in detecting environmental dangers and in testing the effects of hypoxia, drugs, and photodynamic therapy on cancer cells in a realistic setting.

Tumor-on-chip platforms are becoming major tools since they can control delicate aspects of the tumor microenvironment in a tiny system. Tissues in these devices are built via 3D culture, blood vessels that can provide nutrients, and a design that matches real organs, so they resemble the body more than 2D tests do. Recently, a study conducted by Song et al. [139] illustrated this way of working by embedding a 3D microvascular network in a microfluidic chip to examine the extravasation of tumor cells. It is possible to determine with lasers how extravasation events change when the oxygen supply varies, demonstrating that blood hypoxia causes a rise in breast cancer extravasation through the action of HIF-1α. By turning off HIF-1α under hypoxia, the platform demonstrated the ability to identify key details of cellular processes. Figure 6 (B), which was adopted in this context, visually depicts the setup and quantification of tumor cell behavior within the engineered microvascular network, highlighting its potential as a preclinical model for investigating metastasis and testing anti-invasive therapies.

Microfluidic tumor-on-chip platforms have emerged as powerful tools for mimicking the structural and functional complexity of the tumor microenvironment in vitro. These systems leverage microscale fluid dynamics to recreate physiological conditions such as interstitial flow, nutrient gradients, and mechanical cues within three-dimensional tumor constructs. A notable example is the development of droplet-based microfluidic chips that enable the compartmentalization of reactions and real-time monitoring of biological activity. These chips can precisely control the generation and flow of nanoliter-sized droplets that act as isolated bioreactors, facilitating high-throughput and parallel analysis of tumor responses to environmental factors or treatments. The integration of optical biosensing with luciferase-based bioluminescent systems further enhances their utility by enabling non-invasive, real-time detection of biochemical changes in response to toxins or therapies. In the study by Yakimov et al. [137], a droplet microfluidic chip was engineered to produce 0.1 µL droplets encapsulating bacterial luciferase and cofactors, allowing highly sensitive detection of copper ions as model pollutants. The experimental layout, illustrated in Fig. 6 (adopted from the original publication), demonstrates the chip’s architecture, fluid flow regulation, and photomultiplier-based luminescence detection system. This technology highlights the potential of tumor-on-chip systems not only in environmental sensing but also in biomedical applications, such as evaluating cancer cell responses to hypoxia, drugs, or photodynamic therapy in a dynamic and physiologically relevant microenvironment (Fig. 6 (C)).

## Functional characterization of exosomes from TNBC 3D models

### Tumor progression and metastasis

Exosomes strongly affect the progression and spread of triple-negative breast cancer. Exosomes produced by TNBC cells include oncogenic miR-155 and miR-9, as well as Rab27a and annexin A2, which contribute to EMT, increase the formation of new blood vessels, and breakdown the barrier of blood vessels, which promotes metastasis. One example shows that exosomal miR-155 can block PTEN and DUSP14, which helps tumors by increasing their migration, invasion, and new blood vessel growth [140]. Similarly, annexin A2 carried in serum exosomes has been associated with high-grade TNBC and increased angiogenesis, especially among African-American women [141]. Additionally, Rab27a, a key regulator of exosome secretion, is implicated in metastatic efficiency by facilitating the export of tumor-suppressor miRNAs such as miR-23b, thereby sustaining the invasive phenotype of metastatic cells [142].

Cultures grown as 3D spheroids or organoids have demonstrated that exosomes are more successful in moving their cargo and signaling others to support metastasis than are cultured cells in 2D form. For example, in OSCC models, 3D tumor spheroids show increased resistance and similar features to those of cancer stem cells, and this resistance is reversed by exosomal delivery of a miR-155 inhibitor, resulting in the development of OSCC spheroids that allow for better cancer studies in vitro [143]. These findings are summarized in Table 4, which outlines key studies investigating how TNBC-derived exosomes and their cargo contribute to tumor progression, EMT, angiogenesis, and metastasis across various experimental models.

**Table 4:** Summary of key studies investigating exosomal cargo in TNBC and other cancers driving tumor progression and metastasis

Study	Exosomal cargo	Mechanism of action	Model used	Key findings	Ref
Razaviyan et al. (2024)	miR-155 (inhibited)	Upregulates PTEN/DUSP14; inhibits angiogenesis, migration, invasion	TNBC + HUVEC (2D)	Antagomir-loaded exosomes reverse pro-metastatic traits	[140]
Sayyed et al. (2021)	miR-155 (inhibited)	Reverses EMT and drug resistance via FOXO3a activation	3D OSCC spheroids + xenograft	3D model shows enhanced therapeutic reversal using exosomes	[143]
Chaudhary et al. (2020)	Annexin A2	Promotes angiogenesis and correlates with poor prognosis	Serum exosomes (clinical TNBC samples)	High exosomal AnxA2 in aggressive TNBC, especially in AA women	[141]
Ostenfeld et al. (2014)	miR-23b, Rab27a	Loss of tumor suppressor miRNAs promotes invasion; Rab27 regulates exosome secretion	Bladder cancer cells (isogenic lines)	Rab27 inhibition reduced metastasis; high Rab27B linked to poor prognosis	[142]
Alexander et al. (2017)	miR-155	Regulates immune cell signaling, inflammatory response via SHIP1/IRAK-M	Rab27a/b KO mice	Rab27-dependent exosomes enable inflammatory responsiveness; miR-155 is key cargo	[144]
Xiao et al. (2023)	miR-10527-5p	Inhibits EMT and lymphatic metastasis via Rab10 and Wnt/β-catenin	Esophageal SCC (in vitro + in vivo)	Exosomal miR-10527-5p blocks metastasis and may serve as biomarker	[145]

### Exosomes mediate multidrug resistance

Exosomes are important for causing multidrug resistance (MDR) in various cancers, most notably in breast cancer, where treatment does not work as well because of the genetic and cellular differences in this disease. Because of EVs, drug-resistant traits can be given to sensitive cells, which helps cancer spread and return. Studies have shown that exosomal EphA2 from drug-resistant breast cancer cells plays a major role in encouraging aggressive behavior and increasing the likelihood of spread [146]. In addition, some exosome-transported miRNAs, such as miRNA-100-5P, are involved in cisplatin resistance through mTOR pathway modulation, and miRNA-32-5P in liver and lung cancers is activated by the AKT pathway, revealing how exosomes can influence gene regulation in the development of MDR [147]. In breast cancer, exosomal miRNA-222 facilitates adriamycin resistance, whereas in colon cancer, it resists cetuximab through miRNA-mediated AKT/PTEN pathway signaling [148]. Additionally, researchers have identified p-glycoproteins in exosomes as well as the related proteins MDR3 and ABCG2. These findings point to exosomes as transmitters of drug resistance from one cell to another and serve as important biomarkers and potential targets for cancer therapy. New ways of modeling cancer via 3D techniques are revealing more about how exosomes govern tumor growth, cancer stem cells, and the resistance of cancer to treatment in breast cancer and other diseases [149].

Exosomes are now recognized for their important role in the growth and spread of multidrug resistance (MDR) in cancer, increasing the likelihood of relapse and treatment failure. Compared with healthy people, those with cancer produce a much greater number of exosomes (over 4000 trillion), which may contribute to cancer progression [150, 151]. These exosomes carry bioactive molecules, particularly microRNAs (miRNAs), such as miR-146a-5p, miR-222-3p, miR-151a, and circExo-TRPCS, which function as potent biomarkers for cancer diagnosis and may influence drug resistance pathways [152–154]. Although most previous studies on how exosomes promote cancer resistance have been performed in 2D cultures, the use of 3D breast cancer organoid cultures is a major advance in the exploration of this mechanism. They reproduce the structure of real tumors, so the evaluation of drugs and their effects is more precise. While 3D tumoroids are still being developed, their success in assessing the chemosensitivity of patients with colorectal cancer points to a good future for other cancers [155].

Recent research strongly supports the use of organoids and tumoroids as valuable models to study drug resistance in cancer. These 3D cultures often retain key characteristics of the original tumor, including heterogeneity, making them excellent platforms for personalized drug testing and mechanistic studies. Chai et al. relied on organoid techniques to determine how cancer cells become resistant to drugs, creating organoids that are sensitive and resistant to therapy. They found important genes involved in resistance via genomic and transcriptomic analyses, such as FGFR3 and FBN1. Improved methods for resisting adverse effects, including adapting metabolism, repairing DNA, and slowing the aging process, have been identified. In particular, this study highlighted cancer stem cells as a central part of resistance and proposed ways to fight resistance via organoids, suggesting important results for clinical use [156]. Another study by Fang et al. developed a microarray platform using breast cancer patient-derived organoids (BCOs) to assess resistance to adriamycin. This study demonstrated marked heterogeneity in the drug response between organoid Lines and identified ivermectin as a synergistic agent that enhances adriamycin efficacy. This high-throughput 3D model enables efficient on-chip drug screening and viability assessment, emphasizing the value of organoids for individualized therapy development [157].

## Integration of 3D culture models and chromatography for TNBC exosome profiling enhanced biomarker specificity via 3D-derived exosomes

Traditional 2D cultures often fail to replicate the complexity of the tumor microenvironment, limiting the biological relevance of exosomal biomarkers derived from such systems [158]. In contrast, 3D culture models—such as spheroids, organoids, and patient-derived xenografts—have emerged as superior platforms for exosome production, as they more accurately simulate in vivo conditions, including cell polarity, extracellular matrix interactions, and oxygen gradients. These features significantly influence the composition and function of secreted exosomes, making 3D-derived vesicles more reflective of disease-specific molecular signatures [109].

Thus, the use of techniques such as size exclusion chromatography and immunoaffinity purification leads to the efficient separation and reanalysis of exosomes in 3D cultured media [101]. Integrating these methods has made it possible to find certain biomarkers in TNBC, such as CD151 [159], glypican-1 [160], CLDN7 [161], and circCRIM1 [162], along with miR-103a-3p and miR-423-5p, which strongly match what is observed in patient samples [163]. Importantly, 3D-derived exosomes are more varied in their molecules and react well to factors such as drug-induced stress, indicating that extra care can be taken to identify EMT-related cargo such as the lncRNA DARS-AS1 [164].

Beyond improved analytical clarity, 3D-exosome profiling shows high translational potential by enabling the discovery of noninvasive biomarkers with greater sensitivity and specificity than those derived from conventional methods. This approach has demonstrated stronger alignment with patient-derived serum exosome profiles, offering new opportunities for precision diagnostics and therapy monitoring [108]. The comparative advantages of 3D culture systems over 2D culture systems, such as increased cargo specificity, improved biomarker resolution, and clinical relevance, are summarized in Table 5, which shows a comparison of 2D vs. 3D culture models for TNBC exosome profiling and biomarker discovery via chromatography. Together, the combined use of 3D cultures and chromatography offers a robust, physiologically relevant pipeline for exosome-based biomarker discovery in TNBC.

**Table 5:** Comparison of 2D vs. 3D culture models for TNBC exosome profiling and biomarker discovery via chromatography

Parameter	2D culture models	3D culture models (spheroids, organoids, bioprinted tissues)	Impact on chromatographic profiling	Relevance to TNBC biomarker discovery
Cell‒cell & cell–matrix interactions	Poorly mimics tumor microenvironment, lacks stromal interaction	Closely replicates in vivo TNBC tissue architecture, ECM, and gradients	Enhances specificity and separation of biologically relevant exosomes	Improves identification of clinically actionable exosomal biomarkers [88]
Exosome yield & heterogeneity	High yield but often contains nonspecific vesicles and debris	Lower yield but enriched in tumor-specific and functional exosomes	Chromatographic purity and resolution are higher with 3D-derived exosomes	Yields subtype-specific protein and RNA signatures for TNBC [165]
Exosomal cargo (proteins, miRNA, lipids)	Cargo may lack relevance to clinical phenotype; altered due to 2D stress	Mimics physiological cargo; includes EMT-related proteins, miRNAs	Allows precise chromatographic separation based on cargo content	Enables discovery of TNBC-specific targets like CD151, miR-423-5p, circRNAs [166]
Dynamic profiling potential	Static culture limits real-time tracking of changes in exosomal cargo	Captures evolving tumor-secretome dynamics under stress, drugs	More robust proteomic/metabolomic fingerprint via mass-spec chromatography	Facilitates monitoring of treatment resistance and immune escape markers [65, 112]
Translational value	Limited in clinical predictiveness; overrepresents in vitro artifacts	High fidelity to human tumor pathology; supports PDX & patient-derived organoids	Results in exosomes with higher clinical relevance and analyte quality	Boosts predictive value for early detection, prognosis, and therapeutic response in TNBC [167]
Tumor microenvironment simulation	Lacks oxygen/nutrient gradients and cell heterogeneity	Recreates hypoxia, stromal interactions, and nutrient gradients	Exosomes from 3D cultures reflect in vivo TME conditions, improving chromatography resolution	Leads to more physiologically relevant exosomal markers (e.g., CLDN7, CD109) [168, 169]
Molecular diversity of exosomes	Uniform vesicle profiles; misses subtype heterogeneity	Captures intratumor diversity—different exosomes from core vs. periphery	More complex, distinct exosomal populations require refined chromatography (e.g., SEC + affinity)	Improves detection of subtype-specific biomarkers like CD151 and exo-circRNAs [165, 170]
Responsiveness to therapy/stress conditions	Limited modeling of drug resistance mechanisms	Reflects therapy-induced microenvironmental stress (e.g., hypoxia, EMT)	Enables chromatographic profiling of stress-induced exosomal cargo (e.g., miRNAs, lncRNAs)	Supports identification of resistance-related exosomal signals like DARS-AS1, TGF-β miRNAs [171, 172]
Biomarker discovery through integrated omics	Often limited to transcriptomics/proteomics in isolation	Enables integrated multiomics: proteomics + metabolomics + transcriptomics	Chromatography platforms can simultaneously resolve exosomal proteins, lipids, RNAs	Enables robust discovery pipelines for novel diagnostic/prognostic markers (e.g., exosomal Glypican-1, miR-103a) (Igyártó et al., 2018), [168, 173]

### Chromatographic profiling of 3D-derived exosomes in TNBC

The specificity and complexity of exosomes derived from three-dimensional (3D) tumor models make them ideal candidates for advanced chromatographic profiling. Techniques such as size exclusion chromatography (SEC), immunoaffinity chromatography, and ultrahigh-performance liquid chromatography (UHPLC) are increasingly employed to isolate pure exosome subpopulations for downstream proteomic and metabolomic analyses. SEC, in particular, is favored for its ability to gently and efficiently separate exosomes from proteins and other contaminants, preserving their functional integrity. Using these approaches, scientists have identified CD151, Glypican-1, miR-103a, miR-423-5p, and miR-373 biomarkers related to TNBC, which are Generally missed in 2D cultures. Biomarkers of this type help in identifying diseases early, following the effects of therapy and forecasting what will happen to patients [65].

Profiling of plasma EV protein content revealed differences in TNBC patients who were not found in healthy individuals or those with benign breast diseases, suggesting that liquid biopsy may play a role in rapid testing and monitoring of cancer. Furthermore, the proteins, miRNAs, and long noncoding RNAs present in exosomes help reveal the diversity of TNBC and provide many opportunities to find new treatments for each patient. Further research may allow exosome profiling and chromatography to benefit clinical oncology, especially in treating TNBC [174].

The latest studies strongly highlight how 3D culture systems have greatly transformed research on exosomes in TNBC, especially by helping identify chromatographic profiles and molecular features. Unlike 2D cultures, 3D-obtained exosomes better reflect the environment found inside tumors, such as different cell mixtures, interactions between cells and surfaces, hypoxia, and uneven distributions of nutrients [49]. This high compatibility with biological systems makes 3D-made exosomes more useful for finding new biomarkers and performing different tests. Owing to size exclusion chromatography (SEC), immunoaffinity chromatography, and updated ultrahigh-performance liquid chromatography (UHPLC), it is possible to concentrate these high-purity exosomes for effective and accurate proteomic and metabolomic analysis. Studies have shown that H2A, SUMO1P3, and XIST are among the proteins and lncRNAs whose levels are specifically increased in these exosomes and are tied to chances of recurrence and survival issues [49].

Yousafzai et al. [175] reported that three-dimensional culture models help make TNBC-derived exosomes more physiologically relevant. Unlike 2D systems, 3D organoids and spheroids closely resemble tumors in nature and their environments, so the exosomes they produce are a better reflection of real tumors. Through their review, it was emphasized that exosomes produced from 3D cultures are better at promoting communication between cells, especially in regard to resisting drugs, moving through tissue and stimulating the growth of blood vessels. They stated that combining 3D cultures and measuring chromatographic profiles can lead to better identification of biomarkers meant for diagnosis or therapeutics. Another study by Li et al. [165] revealed that the CD151 protein was significantly overexpressed in exosomes taken from the blood of TNBC patients. By using tandem mass tag-based mass spectrometry, CIBERSORT revealed that exosomes containing higher levels of CD151 tend to distribute more ribosomal proteins and fewer complement proteins. The migration and invasion abilities of TNBC cells were reduced when exosomes lacking CD151 were added, which underlines the role of chromatography in highlighting useful exosomal markers. Pullan et al. [176] explored the functional utility of exosomes in drug delivery, specifically engineering bovine milk exosomes with iRGD peptides and hypoxia-sensitive Lipids for targeted doxorubicin delivery. These exosomes demonstrated enhanced cytotoxicity in 3D TNBC spheroid cultures, validating the importance of model choice in drug efficacy studies. Chromatographic methods were used to purify and characterize the exosomes prior to drug loading, confirming the role of purification techniques in functional exosome-based therapeutics.

### Clinical translation of 3D-derived exosome biomarkers in TNBC

The clinical translation of 3D-derived exosome biomarkers in triple-negative breast cancer (TNBC) has advanced rapidly, driven by the unique ability of 3D culture systems to better recapitulate the tumor microenvironment and thus yield exosomes with more physiologically relevant cargo. Compared with cultures on 2D plates, spheroids and organoids in 3D models have allowed scientists to identify key distinctions between the proteins and Genes found in exosomes, providing a better understanding of tumor activity. Owing to their improved specificity, exosome biomarkers extracted from 3D models may provide useful tools for TNBC diagnosis, monitoring patients’ conditions and monitoring treatments [175].

Several clinical studies and trials have shown that proteins and miRNAs attached to exosomes can help identify and predict TNBC. For example, mass spectrometry-based analyses of plasma-derived EVs revealed the protein histone H2A, which is a unique indicator of TNBC and is completely absent under healthy or benign conditions. Similar results have been confirmed both in biopsies from patients and in cell lines, indicating how these biomarkers help identify and manage the disease. In addition, the changes in the exosomal protein cargo before and after surgery suggest their importance for both tracking the results of treatment and detecting the smallest traces of cancer remnants [174].

The therapeutic landscape is also evolving, with 3D-derived exosomes being explored as delivery vehicles for targeted therapies, including miRNA-based interventions. Several studies have demonstrated that exosomes loaded with tumor-suppressive miRNAs can inhibit TNBC cell proliferation, migration, and invasion both in vitro and in vivo. For example, exosome-mediated delivery of miR-424-5p or miR-3182 has been shown to be effective in suppressing tumor growth and modulating immune responses, highlighting the potential for personalized exosome-based therapeutics in TNBC [177].

Despite these promising developments, several challenges remain. The majority of exosome-related clinical trials have focused on biomarker discovery and diagnostic applications, with relatively few addressing therapeutic translation. Standardization of exosome isolation and characterization protocols, validation of biomarker panels in large, diverse patient cohorts, and integration with advanced analytics, such as AI-driven multimarker prediction models, are critical next steps for clinical adoption [178]. Additionally, the scalability and reproducibility of 3D culture systems must be addressed to ensure the consistent production of clinically relevant exosomes.

## Challenges and limitations

### Heterogeneity in exosome subpopulations

One of the foremost challenges in exosome-based research is the biological and molecular heterogeneity of exosome subtypes, particularly in triple-negative breast cancer (TNBC). Exosomes differ widely in size, content, biogenesis pathways, and cell-of-origin, even within a single tumor. This complexity undermines efforts to assign specific biological functions, such as miRNAs, lncRNAs, or membrane proteins, to exosome cargo. For example, exosomes from TNBC subtypes carry distinct transcriptomic signatures, impacting their role in metastasis and immune modulation. Wang et al. (2023) developed a 9-gene exosome-based risk model showing substantial differences in prognosis and immune infiltration between TNBC subgroups, underscoring the need to account for exosomal diversity in both research and clinical applications [169].

To isolate and characterize specific exosome populations accurately, multidimensional separation techniques are increasingly necessary. These methods may involve a combination of ultracentrifugation, size-exclusion chromatography, immunoaffinity capture, and microfluidic-based sorting, each of which has trade-offs in purity, yield, and scalability. Without such stratification, pooled exosome samples may lead to inconsistent or misleading interpretations of therapeutic efficacy or diagnostic accuracy [53].

### Standardization and reproducibility

Ensuring reproducibility among different laboratories is a major challenge because there are no set steps for handling and testing exosomes. Variables, including the cultural environment, makeup of the medium, timepoints for centrifugation, and how one assesses the results, add considerable technical noise. According to Wang et al. (2024), despite high-quality research on exosomes, uniform descriptions and careful checks are not common, making it difficult to compare outcomes or validate biomarkers [179].

The use of 3D culture makes this problem worse, as the number of exosomes is strongly driven by variations in oxygen, pH, and nutrient supply, all of which are affected by how the system is built and packed with cells. Unless there are strong protocols and accepted validation rules, the findings from 3D models of exosomes are not very reproducible. Tzng et al. (2023) reported that for exosome-based therapeutics to be consistently used, stored, absorbed, and accounted for in regulatory guidelines, their handling should be as stringent as that of biologics and nanomedicines [180].

### Scalability of 3D models and chromatographic systems

Translating 3D tumor spheroid/organoid models and exosome-based therapeutics into scalable, clinically feasible platforms remains a formidable barrier. While 3D models better recapitulate in vivo tumor behavior and exosome dynamics, their use is often confined to small-scale research due to technical constraints. Issues include high cost, long culture times, batch-to-batch variability, and limited integration with high-throughput drug screening systems.

In the context of exosome manufacturing, traditional isolation methods (e.g., differential ultracentrifugation) are not viable for industrial or clinical-scale production. However, promising strategies are emerging. Haraszti et al. (2018) demonstrated that 3D microcarrier-based cultures of mesenchymal stem cells, when combined with TFF, produced 20–140 times more biologically active exosomes than did standard 2D protocols. These 3D-TFF exosomes also exhibited enhanced therapeutic RNA delivery efficiency, revealing a path toward scale-up [181].

Despite their promise in modeling TNBC and advancing personalized therapies, exosomes and 3D culture systems face intertwined challenges of heterogeneity, lack standardization, and have limited scalability. Addressing these barriers is essential for translating laboratory findings into clinically impactful interventions for aggressive cancers such as TNBC.

### Limitations in TNBC exosome biomarker research

Despite the promising potential of exosome-derived biomarkers for the early detection, prognosis, and monitoring of triple-negative breast cancer (BC), several key limitations limit their clinical utility. First, the heterogeneity of exosomal cargo driven by both inter- and intratumoral variability complicates the identification of universally reliable biomarkers; distinct TNBC subclones secrete exosomes with differing miRNA and protein profiles, leading to inconsistent diagnostic signatures across patient cohorts [53]. Second, isolation and purity challenges persist: conventional ultracentrifugation and size-exclusion methods often coisolate contaminants (e.g., lipoproteins and protein aggregates) that interfere with downstream analyses, reducing specificity and increasing background noise in detection assays [182]. Third, sensitivity constraints arise because exosomal biomarker concentrations in peripheral blood can be exceedingly low, particularly in early-stage TNBC, necessitating highly sensitive platforms that are not yet standardized for routine clinical use [183]. Fourth, the lack of standardized protocols for exosome collection, storage, and analysis leads to significant variability in reported biomarker performance and hinders cross-study comparisons. Finally, translational and regulatory hurdles—including the need for large, multicenter validation studies, reproducible manufacturing of exosome-based assays, and compliance with stringent quality controls—remain barriers to the seamless integration of TNBC exosome biomarkers into clinical practice. Collectively, these limitations underscore the need for rigorous standardization of exosome workflows, development of ultrasensitive and high-throughput detection technologies, and comprehensive validation in well-characterized patient populations before exosome biomarkers can reliably inform TNBC management.

### Technical Limitations in 3D culture systems

Three-dimensional (3D) cell culture platforms, which offer more physiologically relevant microenvironments than traditional two-dimensional models do, are encumbered by significant technical challenges that undermine reproducibility and translational potential. Foremost among these is the batch-to-batch variability of biological matrices such as Matrigel: proteomic analyses reveal as Little as 50–60% overlap in protein composition between lots and elastic modulus values ranging from ~ 400 to 840 Pa, with local “hotspots” up to 1–3 kPa, driving unpredictable cell differentiation, morphogenesis, and drug responses [184]. In combination, handling and automation hurdles arise from the high viscosity and thermosensitive gelation kinetics of ECM hydrogels: rapid polymerization at ambient temperatures leads to pipette clogging, whereas standard air-displacement Liquid-handling systems exhibit poor precision at volumes below 10 µL, impeding high-throughput workflows and exacerbating volume and seeding density inconsistencies [185]. Within the gel, mass-transport constraints yield steep nutrient, oxygen, and waste gradients, and oxygen diffusion seldom exceeds ~ 150 µm, creating hypoxic cores in larger constructs and nonuniform reagent penetration that skews viability assays and biochemical measurements. Imaging and quantification further suffer: the acquisition of volumetric datasets via confocal or light-sheet microscopy generates tens of gigabytes per plate, and cell retrieval from dense matrices for single-cell analyses remains both inefficient and variable, whereas the absence of standardized image-analysis pipelines thwarts high-content screening adoption. Finally, the lack of consensus standards for scaffold composition, stiffness calibration, and seeding protocols across laboratories perpetuates irreproducibility, as slight differences in vendor, lot, or dispensing technique can drastically alter experimental outcomes; although chemically defined synthetic hydrogels promise tunable mechanics and reduced variability, they often lack critical ECM-derived biochemical cues, necessitating complex biofunctionalization to support faithful tissue-specific behavior [184, 186]. Addressing these interlinked Limitations requires a concerted effort to develop defined, reproducible matrices, optimize automation for viscous biomaterials, and establish community-wide quality control and analysis standards to harness the full potential of 3D culture systems.

### Standardization issues in exosome isolation and clinical translation

The clinical deployment of exosome-based assays is severely hampered by inconsistent isolation protocols and insufficient quality control standards. First, protocol heterogeneity ranging from ultracentrifugation and size-exclusion chromatography to polymer-based precipitation and immunoaffinity capture yields exosome preparations with widely variable purities, yields, and coisolated contaminants (e.g., lipoproteins and protein aggregates), making cross-study comparisons unreliable [187]. Second, patient-to-patient variation in exosome abundance and composition arises from differences in disease state, comorbidities, and sample handling, complicating the establishment of universal biomarker thresholds and reducing diagnostic sensitivity and specificity [188]. Third, immunogenicity concerns stem from residual nonexosomal proteins, particularly when biological matrices are used for cell culture or to capture antibodies for isolation, which can elicit unwanted immune responses upon administration or skew assay readouts [100]. Finally, regulatory ambiguity persists due to the lack of consensus on critical quality attributes (particle size distribution, protein markers, and RNA content) and acceptable analytic validation metrics, delaying the formulation of harmonized guidelines for manufacturing, release testing, and clinical qualification [189]. Overcoming these challenges will require the development of validated, high-throughput isolation platforms; the establishment of standardized minimal information reporting frameworks; and multicenter studies to define robust, reproducible exosome quality–control criteria that ensure safety and efficacy in diverse patient populations.

## Translational gaps and future directions

Despite significant technological progress, the clinical translation of 3D culture models and advanced chromatography techniques in exosome research for TNBC remains constrained by critical systemic and scientific challenges. While 3D culture models are more physiologically relevant than 2D systems are, they suffer from poor standardization and reproducibility, especially in large-scale studies or multicenter trials. Variability in scaffold materials, cell seeding density, and matrix composition leads to inconsistencies in exosome output and behavior, making comparative studies and regulatory assessments extremely difficult [190]. Moreover, although they better mimic the TNBC tumor microenvironment, most 3D models lack immune and vascular components, which are critical for evaluating exosome-mediated communication in vivo. This limits their ability to predict clinical outcomes.

Advanced chromatography techniques—such as ultracentrifugation combined with size exclusion or affinity purification—have improved the isolation and purity of exosomes. However, these methods often require highly skilled personnel and specialized equipment and are not yet scalable for clinical-grade good manufacturing practice (GMP) conditions [191]. These approaches also struggle to preserve the functional and structural integrity of exosomes, which is essential for their use as therapeutic agents or biomarkers. Furthermore, regulatory bottlenecks persist—there is no consensus from the FDA or other agencies on what constitutes clinically relevant exosome characterization standards. This includes variability in quantification, cargo profiling, and functional validation. As a result, many studies remain preclinical or exploratory, with a limited transition to investigational new drug (IND) applications or early-phase clinical trials [192].

A particularly overlooked challenge is patient-specific variability. TNBC is highly heterogeneous, and exosome profiles vary widely between individuals and even within tumors over time. Personalized applications, such as the use of exosomes for targeted drug delivery or real-time disease monitoring, require high-throughput, real-time exosome analysis tools that can adapt to dynamic changes in tumor biology. These tools are currently lacking, and existing methods are too slow, expensive, and technically fragile for routine clinical use [193].

In future directions, a major priority must be the convergence of microfluidics, AI-driven pattern recognition, and regulatory-grade manufacturing to bridge these gaps. For example, integrating 3D models with lab-on-chip exosome collection and analysis platforms may help overcome both scalability and real-time monitoring hurdles. Moreover, codevelopment with regulatory agencies is essential to create adaptive frameworks for approving complex, modular, and personalized exosome-based diagnostics and therapeutics.

## Conclusion

Triple-negative breast cancer (TNBC) remains one of the most aggressive and therapeutically challenging subtypes of breast cancer. This review underscores the pivotal role of exosomes in TNBC progression, metastasis, immune modulation, and drug resistance. By integrating advanced chromatographic techniques with physiologically relevant 3D culture models, including spheroids, organoids, hydrogel scaffolds, bioprinted constructs, and microfluidic tumor-on-chip platforms, researchers can now study exosome biology with greater resolution, fidelity, and translational relevance. The synergistic application of high-performance separation methods (e.g., SEC, HPLC, and immunoaffinity chromatography) with 3D systems has enabled a more accurate representation of the tumor microenvironment and improved the functional characterization of exosome subpopulations. These methodological advances are crucial for discovering novel biomarkers, improving therapeutic targeting, and overcoming multidrug resistance in TNBC. The future of exosome-based diagnostics and therapeutics in TNBC will depend on the continued refinement of these interdisciplinary platforms, incorporation of multiomics analyses, and eventual clinical validation through patient-derived models. Bridging the gap between in vitro modeling and in vivo application will be essential to realize the promise of precision oncology for TNBC patients.

## Supplementary Information

Supplementary Material 1.

## Data Availability

All the data generated or analyzed are included in this article.

## Abbreviations

TNBC: Triple-negative breast cancer
sEVs: Small extracellular vesicles
ECM: Extracellular matrix
TME: Tumor microenvironment
EMT: Epithelial-to-mesenchymal transition
CSC: Cancer stem cell
dECM: Decellularized extracellular matrix
SEC: Size-exclusion chromatography
AF4: Asymmetrical flow field-flow fractionation
LC‒MS: Liquid chromatography‒mass spectrometry
SERS: Surface-enhanced Raman scattering
HER2: Human epidermal growth factor receptor 2
ER: Estrogen receptor
PR: Progesterone receptor
miRNA: MicroRNA
lncRNA: Long noncoding RNA
siRNA: Small interfering RNA
DNA: Deoxyribonucleic acid
RNA: Ribonucleic acid
MVB: Multivesicular body
AR: Androgen receptor
BL1: Basal-like 1
BL2: Basal-like 2
M: Mesenchymal
IM: Immunomodulatory
LAR: Luminal androgen receptor
PARP: Poly (ADP‒ribose) polymerase
