# Supplementary figures and images for: The role of 3D culture models and advanced chromatography in exosome research for triple-negative breast cancer
Source: J Egypt Natl Canc Inst. 2025 Sep 27;37:67. doi: 10.1186/s43046-025-00322-x (PMC13313435; doi:10.1186/s43046-025-00322-x)

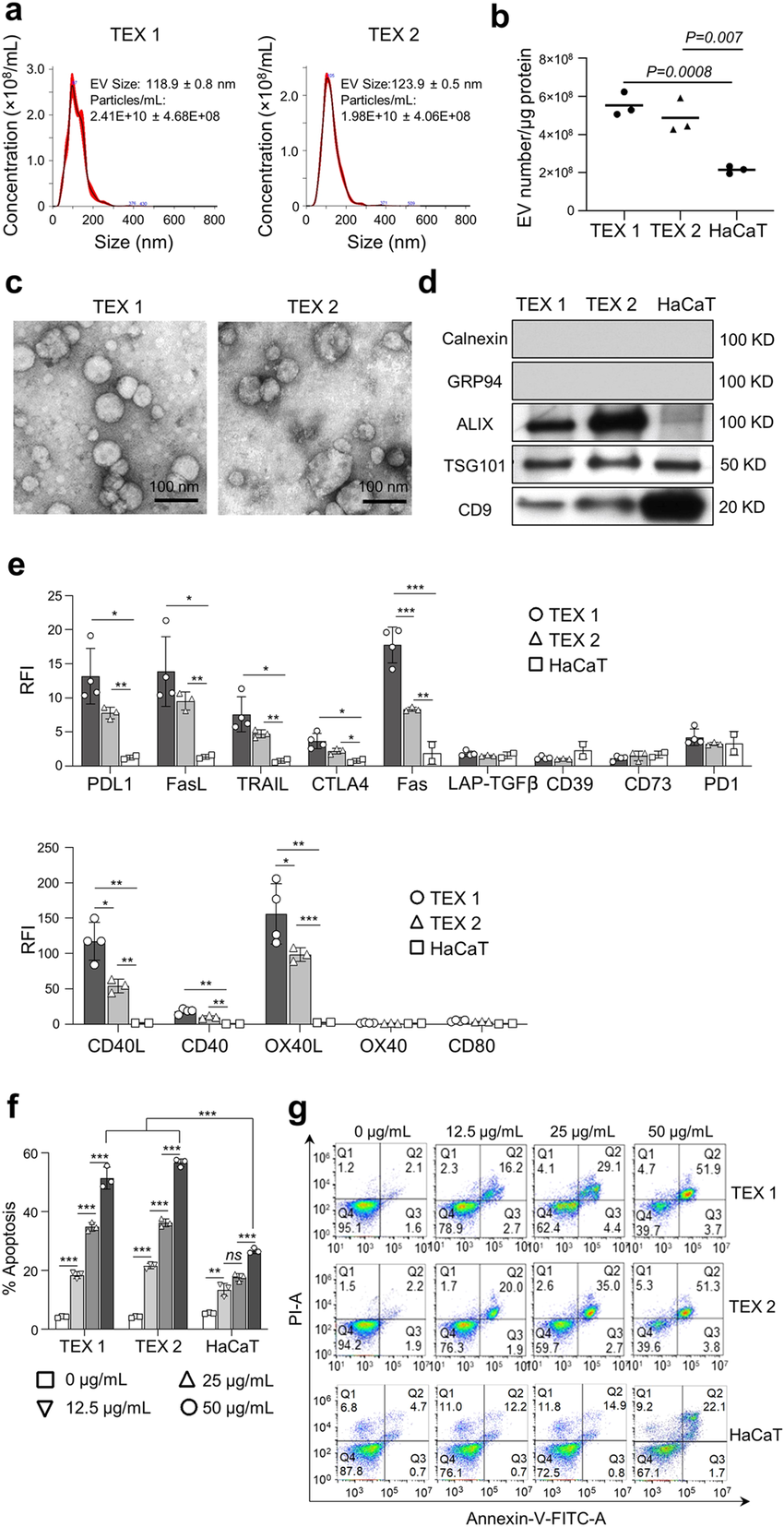

Supplement: Supplementary file 1 — Supplementary Material 1. [file 43046_2025_322_MOESM1_ESM.png]
